# Voxelwise structural disconnection mapping: Methodological validation and recommendations

**DOI:** 10.1016/j.nicl.2022.103132

**Published:** 2022-07-29

**Authors:** Max Wawrzyniak, Anika Stockert, Julian Klingbeil, Dorothee Saur

**Affiliations:** Neuroimaging Laboratory, Department of Neurology, University of Leipzig Medical Center, Leipzig, Germany

**Keywords:** Stroke, Disconnection, Structural connectivity, BCBtoolkit, Dti, Hemiparesis, Corticospinal tract, CST, corticospinal tract, DWI, diffusion weighted imaging, FSL, FMRIB Software Library, NIHSS, National Institutes of Health Stroke Scale, VLSM, voxel-based lesion-symptom mapping

## Abstract

Voxelwise disconnection mapping is a novel approach to disclose lesion-symptom relationships for symptoms caused by white matter disconnection. It uses MRI-based fiber tracking in healthy subjects seeded from patient’s focal brain lesions. Resulting individual disconnection maps can then be statistically associated with symptoms. Despite increasing use in the recent years, the validity of this approach remains to be investigated. In this study, we validated both, our own implementation and the implementation provided within BCBtoolkit. For technical validation, we used simulated symptoms based on overlap of 70 real stroke lesions with tracts from a white matter atlas. For clinical validation, paresis scores and lesions from 316 patients with stroke were used. We found that voxelwise disconnection mapping is technically valid and outperforms the standard voxel-based lesion-symptom mapping approach for symptoms caused by white matter disconnection. Supporting its clinical validity and utility, we were able to reproduce the known association between corticospinal tract damage and contralateral hemiparesis. In addition, we demonstrate that the validity can be substantially diminished by relatively minor methodological changes. Based on these results, we derive methodological recommendations for the future use of voxelwise disconnection mapping. Our study highlights the importance of validating novel methodological approaches in the rapidly evolving field of neuroimaging.

## 1 Introduction

Focal damage to different eloquent cortical brain areas, e.g., following stroke, can cause specific neurological or neuropsychological symptoms (Broca, 1861, Damasio and Damasio, 1989). Given the brain is organized in networks of interconnected brain regions (Mesulam, 1990), other mechanisms beyond direct lesion effects may additionally account for specific symptoms. First, symptoms may be attributed to undamaged regions functionally or structurally connected to the lesion site. This phenomenon is referred to as diaschisis, in which dysfunction is indirectly caused by missing neural input from another brain area (Carrera and Tononi, 2014, von Monakow, 1914). Second, symptoms may follow disconnection caused by damage to white-matter fiber pathways between brain areas that perform a function in a common cortical/subcortical network (Geschwind, 1965).

The first mechanism, i.e. eloquent damage, implies a direct relationship between lesion site and symptom. To understand structure–function relationships in the brain, attribution of symptoms to lesion locations thus has long been an important tool (Broca, 1861, Damasio and Damasio, 1989), which continues to be used as voxel-based lesion-symptom mapping (VLSM, Bates et al., 2003, Karnath et al., 2018). However, for symptoms caused by diaschisis or disconnection, there is no such close relationship between lesion site and symptom (Boes et al., 2015, Chung et al., 2004). The utility of VLSM for such symptoms therefore is limited.

For diaschisis, this limitation has recently been addressed by the approach of lesion-network mapping (Boes et al., 2015, Fox, 2018). Normative functional connectome data is used to calculate networks of regions functionally connected to the lesion. These lesion networks can then be statistically related to a symptom of interest (Wawrzyniak et al., 2018).

For disconnection, the exact localization of lesions along the affected white-matter fiber pathways is irrelevant for the elicitation of symptoms. The corticospinal tract (CST) is an illustrative example: lesions at any location along this tract (e.g., periventricular white matter, internal capsule, midbrain, pons, medulla oblongata, spinal cord) can cause central hemiparesis (e.g. Feng et al., 2015). VLSM analyses may remain silent in datasets containing a variety of different lesion locations along this tract with little or no overlap at all. This limitation can be addressed using normative structural connectome data. Fiber tracking seeded from individual lesions could implicate a specific tract in all lesions at any site along this tract allowing to identify the common neural substrate of a symptom caused by disconnection. Importantly, this method relies on normative connectome data obtained from publicly available datasets and no specialized imaging other than the image needed for patients’ lesion delineation is necessary.

The first methodological proposal for voxelwise disconnection mapping has been made by Foulon and colleagues (Foulon et al., 2018). This approach is available within the BCBtoolkit (https://www.toolkit.bcblab.com) and has been increasingly used in the recent years (e.g. Alves et al., 2021, Pacella et al., 2019, Salvalaggio et al., 2020, Wiesen et al., 2020). It relies on deterministic fiber tracking seeding from individual lesions (Thiebaut de Schotten et al., 2011). These tractograms are used to generate visitation maps (fibers per voxel) which are then binarized based on a threshold of > 0 fibers on an individual basis. These individual binary maps are summed up across the entire normative cohort resulting in an overlap ranging from 0 % to 100 %. In some studies, this map is again thresholded at ≥ 50 % (e.g. Alves et al., 2021, Foulon et al., 2018, Wiesen et al., 2020). The result is one disconnectome map per patient. Second level statistics in terms of an association with a symptom of interest are performed either using an algorithm called AnaCOM (Foulon et al., 2018), within the framework of the general linear model using permutation tests and threshold-free cluster enhancement (Alves et al., 2021, Monai et al., 2020, Pacella et al., 2019) or using multivariate methods (Salvalaggio et al., 2020, Weaver et al., 2021, Wiesen et al., 2020). An alternative to the voxelwise approach is to examine disconnection on the level of macroscopic tracts from white matter atlases (Foulon et al., 2018). We here focus on the voxelwise approach because of its higher spatial resolution and its independence from choice of white-matter atlas.

Since its first release, voxelwise disconnection mapping has been used increasingly for a variety of different symptoms, e.g. semantic fluency deficits (Foulon et al., 2018), anosognosia for hemiplegia (Monai et al., 2020, Pacella et al., 2019), post-stroke depression (Weaver et al., 2021) or prediction of multimodal deficits after stroke (Salvalaggio et al., 2020). Although the method has produced plausible results, both, technical and clinical validation are an important premise for future use. Potential interference could result from heteroscedasticity in modelling errors when using general linear models due to the thresholding of the disconnectome maps (i.e. keeping values ≥ 50 % and discarding values < 50 %) performed in some studies and enabled by default in BCBtoolkit (Foulon et al., 2018). This might lead to inflated FWE-rates in the permutation tests often used in the second level analyses (Huang et al., 2006). In addition, the algorithm of AnaCOM (integrated in BCBtoolkit) might offer poor specificity (Rorden et al., 2009). The only validation of this method to date is based on 38 patients with brain lesions of various etiologies with regard to semantic fluency deficits. Results obtained with AnaCOM are compared to automatic fMRI meta-analyses for the terms ‘fluency’ and ‘category’ (Foulon et al., 2018). No further attempts have been made to technically or clinically validate voxelwise disconnection mapping and its results. Therefore, although increasingly used over the recent years, the validity and clinical utility of structural disconnection mapping (and their methodological conditions) need further exploration.

In the current study we first aimed to establish a voxelwise disconnection mapping approach based on probabilistic tractography addressing some of the mentioned methodological constraints. Second, we aimed to technically and clinically validate our own approach and the implementation in BCBtoolkit. We used (i) simulated data of atlas-based tract damage and (ii) real data regarding hemiparesis following stroke. Finally, we discuss the potential scientific and clinical utility of the approach in relation to methodological details and make recommendations for best practice.

## 2 Material and methods

We performed two main analyses to which we refer to throughout the manuscript as ‘analysis 1’ (technical validation) and ‘analysis 2’ (clinical validation).

**Fig. 1:**
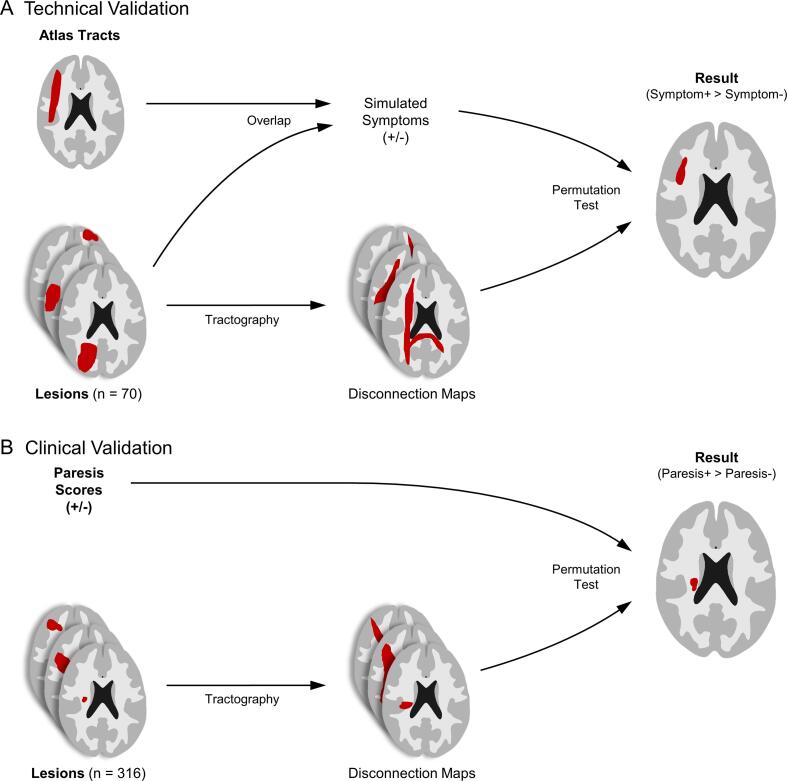
**Methodological overview.** Illustration of (A) technical and (B) clinical validation. See text for detailed explanation.

For analysis 1 (technical validation, Fig. 1A), we performed a modified voxelwise disconnection mapping (described in detail below) and the established approach available with BCBtoolkit (Foulon et al., 2018). In both approaches, we used 70 real stroke lesions taken from a prior lesion study (Wawrzyniak et al., 2018, Zeller et al., 2011) and simulated behavioral data. In analogy to VLSM validation studies (Mah et al., 2014, Sperber and Karnath, 2017), a tract-specific symptom was assumed to be present in patients whose lesion overlapped with a specific tract in the JHU white-matter tractography atlas (Wakana et al., 2007). We then tested for differences in the disconnection maps between patients with and without a simulated symptom expecting to find the same white-matter tracts used to simulate the symptom. This intended circularity allows us to technically validate the different approaches.

Analysis 2 (Fig. 1B) aimed to clinically validate the approach based on 316 stroke patients from another prior lesion study (Klingbeil et al., unpublished) for whom paresis scores one week after stroke were available. It is established neuroanatomical knowledge that hemiparesis after stroke is caused by damage to the CST (e.g. Feng et al., 2015). The approach of voxelwise disconnection mapping would therefore prove clinically relevant if the CST could indeed be reproduced when testing for differences in the disconnection maps between patients with and without hemiparesis.

### 2.1 Patients and data

All experimental procedures were approved by the local ethics committees according to the Declaration of Helsinki. Written informed consent was given by each participant or her/his legal guardian.

#### 2.1.1 Technical validation cohort

**Fig. 2:**
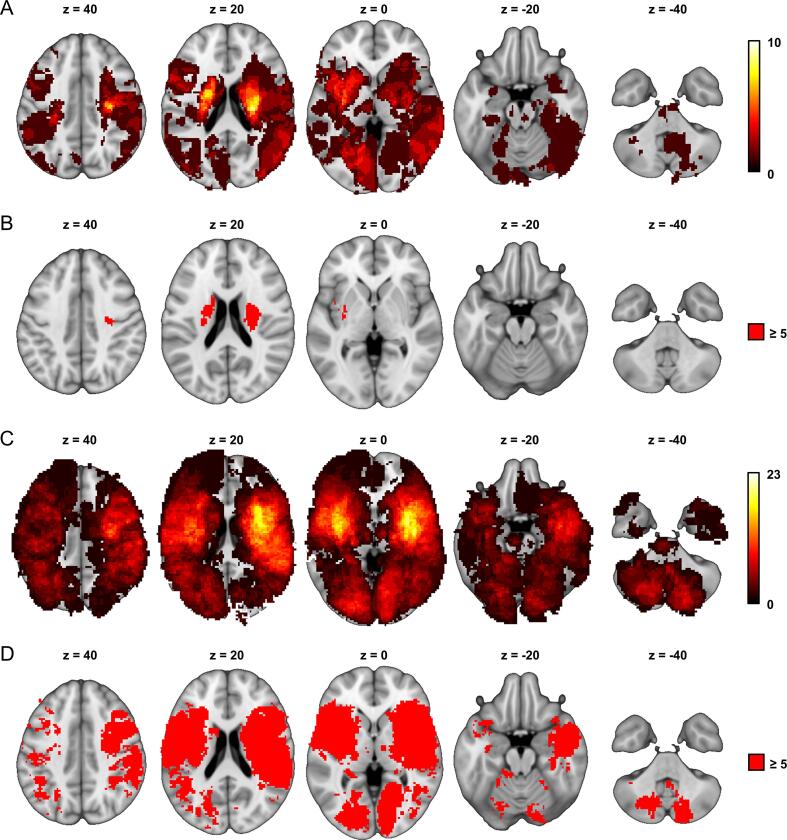
**Lesion characteristics.** Lesion overlap of (A) the technical validation cohort and (C) the clinical validation cohort are shown in warm colors. Regions with sufficient overlap for VLSM (n ≥ 5) in (B) the technical validation cohort and (D) the clinical validation cohort are displayed in red. Coordinates refer to MNI space. Left hemisphere is displayed left. (For interpretation of the references to colour in this figure legend, the reader is referred to the web version of this article.)

We used lesion masks from a prior study with 70 consecutive patients (aged 21–86, mean age 59.5) with a first-ever, acute (1–7 days after onset) focal ischemic unilateral left (n = 37) or right (n = 33) sided brain lesion. Individual lesion masks were delineated based on diffusion-weighted imaging (DWI) (0–14, mean 4.3 days after stroke onset). Spatial normalization to the MNI template was performed with the FMRIB Software Library (FSL, Jenkinson et al., 2012). See Fig. 2A for lesion overlay.

#### 2.1.2 Clinical validation cohort

Lesion masks and paresis subscores from the NIHSS (National Institutes of Health Stroke Scale) for 316 patients (aged 18–89, mean age 66.8, 59.8 % male) were taken from another prior study on post-stroke depression (Klingbeil et al., unpublished). Lesions were delineated on clinical imaging in terms of MRI (∼75 %) or CT (∼25 %) scans obtained between days 0 and 44 (mean 2.7). Stroke symptoms were scored about one week after stroke onset (day 1–27, mean 6.1). Normalization to MNI space was performed using the Clinical Toolbox (Rorden et al., 2012) for SPM12 (Wellcome Trust Centre for Neuroimaging, London, United Kingdom). See Fig. 2C for lesion overlay.

#### 2.1.3 Normative structural connectome data

We used diffusion weighted MRI scans (2x2x2 mm, 128 directions, b-value 1500 s/mm^2^ and additional nine b0-weighted images) and T1-weighted MPRAGE images (1x1x1 mm, TR/TE: 1900/2.52 ms, flip angle: 9°) of 187 healthy subjects aged 18 to 84 years from the Enhanced NKI sample (Nooner et al., 2012).

### 2.2 Data analysis

#### 2.2.1 Fiber tracking

Fiber tracking including preprocessing was performed with FSL v6.0. Default parameters were used if not otherwise stated. All diffusion data was corrected for eddy current-induced distortions and subject movements (Andersson and Sotiropoulos, 2016) and non-brain tissue was deleted (Smith, 2002). We then performed bayesian estimation of diffusion parameters obtained using sampling techniques (Behrens et al., 2007, Jbabdi et al., 2012). A series of linear (DWI to T1-weighted image, T1 to MNI template) and non-linear (T1 to MNI) spatial registrations (Jenkinson and Smith, 2001) and their inversion was performed to transform regions of interest into the native DWI space and tractography results into MNI space.

Native space probabilistic tractography as implemented in ‘probtrackx2′ was seeded from individual lesion masks. Path distributions were corrected for the length of the pathways, divided by the total number of generated fibers (to account for differently sized seed masks) and then stored in MNI space for further processing.

Because the probabilistic tractography is computationally very expensive, we decided to work with a subset of DWI datasets instead of performing full tractography in all 187 healthy subjects for every lesion. To this end, we performed tractography in all 187 patients for four random lesions from the technical validation cohort. We calculated correlation coefficients between mean connectivity of differently sized random subsets and mean connectivity of all remaining subjects 1,000 times. This enabled us to plot shared variance (R^2^, mean and 95 % confidence interval) against the number of used DWI datasets (SI Fig. 1A). We found that random sets of n = 25 fiber tractograms were sufficient to explain over 90 % of variance. In a second step, we performed the same analysis with regard to age effects (with a fixed size of n = 25) and found high values (i.e. > 90 %) for shared variance in a wide range of age with a slight drop for subjects aged ≥ 65 (SI Fig. 1B). To roughly match stroke patients, we selected 25 healthy subjects (10 females and 15 males) aged 49–64 (mean 58.4) for all further analyses.

#### 2.2.2 Disconnection maps

**Fig. 3:**
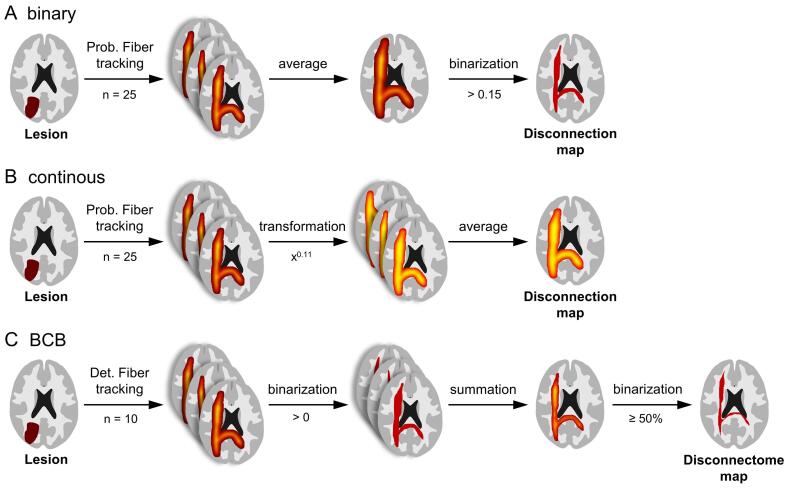
**Illustration of the different disconnection mapping approaches.** Different approaches were used to generate disconnection maps from individual lesions. (A) “binary” approach: probabilistic tractography (n = 25), averaging and binarization using different cutoffs in the technical validation cohort and a cutoff of > 0.15 in the clinical validation cohort. (B) “continuous” approach: probabilistic tractography (n = 25), power transformation (λ = 0.11) and averaging. (C) “BCB” approach: deterministic fiber tracking (n = 10) as implemented in BCBtoolkit, binarization of the individual tractograms, summation and again binarization on the group level (typically at ≥ 50 %, but also using different thresholds in a supplementary analysis).

The raw path distribution maps were highly skewed towards small values (SI Fig. 2A) and therefore not amenable for permutation testing (Huang et al., 2006). We used two methods in separate analyses to address this issue: (i) We calculated the mean across all 25 healthy subjects and then binarized the resulting disconnection map (Fig. 3A). The optimal cutoff value was determined in the technical validation cohort based on visual inspection and Dice coefficents of the results obtained with five different cutoff values. (ii) We applied a power transformation to stabilize variance (Box and Cox, 1964) prior to calculating the mean and refrained from additional binarizing (Fig. 3B). The suitable exponent λ for this transformation was estimated based on path distributions in the technical validation cohort. We iteratively searched for the exponent, which minimized the sum of squared errors between the transformed data (masked by a white-matter skeleton) and a normal distribution with the same mean and standard deviation. We found that variance was best stabilized with an exponent of λ = 0.11 and subsequently transformed all path distribution maps (SI Fig. 2B). Of note, we refrained from binarizing individual tractograms in both approaches to avoid omitting potentially relevant variance in the data.

Additionally, we calculated disconnectome maps using BCBtoolkit based on DWI data from 10 healthy participants provided with the toolkit (Foulon et al., 2018). These maps were binarized (in contrast to thresholding, which is the default setting in BCBtoolkit) at an overlap of ≥ 50 % (Fig. 3C), which is in analogy with prior studies (Foulon et al., 2018, Monai et al., 2020, Weaver et al., 2021, Wiesen et al., 2020).

#### 2.2.3 Second level analyses

To examine the relationship between symptoms and disconnection maps, we tested for differences between disconnection maps from patients with and without the (simulated or real) symptom of interest. The analyses were performed within the framework of general linear models. Lesion size was included in the model as a covariate of no interest. Significance was assessed by 4,000 random permutations of the design matrix using the Freedman-Lane procedure (Freedman and Lane, 1983) to obtain the critical threshold corresponding to p(FWE) < 0.05 on the voxel-level (Nichols and Holmes, 2002). In the case of binarized disconnection maps, analyses were restricted to voxels with an overlap of at least n = 5. For reference, we also performed VLSM analyses with the same parameters.

### 2.3 Analysis 1 – Technical validation

Disconnection maps for all 70 patients of the technical validation cohort were tested for differences related to simulated symptoms. We used the JHU white-matter tractography atlas (Wakana et al., 2007) to simulate tract-specific symptoms for all 20 available tracts. A symptom was assumed to be present when there was an overlap between the patient’s lesion and the tract (defined as an overlap ≥ 4x4x4 voxels or 0.64 ml with a tract probability of > 25 %). By using absolute damage instead of proportion damaged, we account for the highly anisotropic properties of the examined fiber tracts. Finally, we tested for group differences between disconnection maps of patients with vs. without the symptom for every tract with at least four patients with a simulated symptom. These analyses had an intended circularity (Fig. 1A) and were expected to reveal the same tracts from the JHU atlas as used to simulate the symptoms.

The analyses were performed with disconnection maps binarized with cutoffs of 0.025, 0.05, 0.10, 0.15 and 0.20 as well as with power-transformed (λ = 0.11) disconnection maps and disconnectome maps obtained with BCBtoolkit.

To assess similarity between the disconnection mapping and VLSM results (thresholded at p(FWE) < 0.05) and the tracts used to simulate the symptoms (thresholded at ≥ 25 %), we calculated Dice coefficents between these maps (Dice, 1945).

### 2.4 Analysis 2 – Clinical validation

This analysis aimed to evaluate the clinical utility of disconnection mapping. We relied on the symptom hemiparesis after stroke which is known to be associated with contralateral CST damage (e.g. Feng et al., 2015). We used lesion masks and paresis scores from the NIHSS of 316 stroke patients from the clinical validation cohort. Patients were assigned to the group of left/right hemiparesis if at least one of the following NIHSS items was scored > 0: motor arm, motor leg or facial palsy on the respective side. We tested for differences in the disconnection maps between patients with a left sided hemiparesis and all remaining patients (with no or right sided hemiparesis) and right sided hemiparesis and all remaining patients separately.

The analysis was performed with disconnection maps binarized with a cutoff of 0.15 as this value provided the best results in the technical validation cohort (see below) and with disconnectome maps obtained with BCBtoolkit.

To assess similarity between the disconnection mapping and VLSM results (thresholded at p(FWE) < 0.05) and the CST contralateral to the paresis, we again calculated Dice coefficents between these maps.

### 2.5 Data availability

Lesions in MNI space of the technical and clinical validation cohort as well as group assignment (left/right/no hemiparesis) of the clinical validation cohort are publicly available here: https://doi.org/10.6084/m9.figshare.19536202. All used software packages are referenced in the methods section. Permutation tests were performed based on adapted niiStat’s (https://www.nitrc.org/projects/niistat/) core functions.

## 3 Results

### 3.1 Analysis 1 – Technical validation

Six tracts from the JHU white-matter tractography atlas did not have sufficient overlap with the lesions from the technical validation cohort and therefore had to be excluded. The remaining 14 tracts could be utilized to simulate tract specific symptoms based on lesion overlap with the tract. We performed eight analyses per tract: ordinary VLSM for reference, binarized disconnection mapping with five different cutoffs for binarization (Fig. 3A), disconnection mapping with power-transformed maps (λ = 0.11, Fig. 3B) and disconnectome mapping using BCBtoolkit binarized at ≥ 50 % overlap (Fig. 3C).

**Fig. 4:**
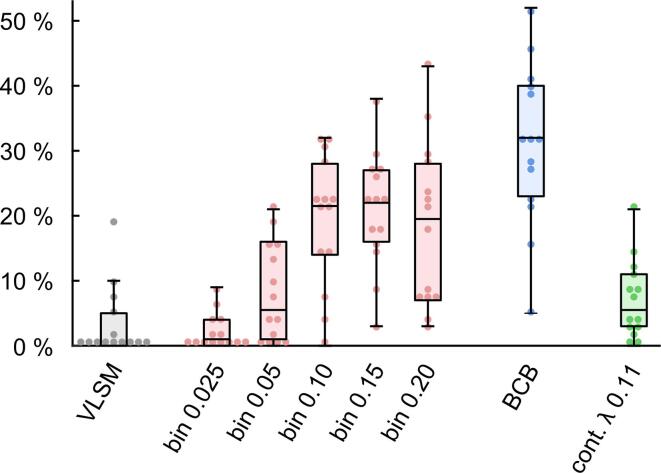
**Dice coefficients – technical validation.** This boxplot displays similarity between the 14 atlas tracts used to simulate symptoms and the results from the different analyses using Dice coefficients. Higher values imply higher similarity. Central marks of the box represent the median value, the edges are the 25th and 75th percentiles, and the whiskers extend to the most extreme data points not > 150 % of the interquartile range beyond the boxes. Filled circles represent Dice coefficients for individual tracts. Abbreviations: VLSM: voxel-based lesion-symptom mapping (grey), bin 0.x: disconnection mapping based tractograms binarized at different thresholds (red), BCB: disconnectome mapping based on tractograms obtained with BCBtoolkit and binarized at 50 % overlap (blue), cont. λ 0.11: continuous disconnection maps which were power transformed with λ = 0.11 (green). (For interpretation of the references to colour in this figure legend, the reader is referred to the web version of this article.)

To give an overview, we display Dice coefficients (analysis result vs. true tract) for all tracts and analysis variants in Fig. 4 (and SI Table 1). These coefficients can reach values between 0 % and 100 %. Low values indicate low similarity between the analysis result and the tract used to simulate the symptom, and vice versa for high values. This figure helps to recognize the general patterns found in the data but may not substitute viewing the actual statistical maps. We here demonstrate the overall patterns found in our results based on a representative example (bilateral CST). Statistical maps from all other tracts can be found in the Supplementary Material.

**Table 1:** **Dice coefficents – clinical validation.** This table displays similarity between the CST and results from VLSM and disconnection mapping analyses regarding contralateral hemiparesis in 316 stroke patients. Higher values imply higher similarity. Abbreviations: VLSM: voxel-based lesion-symptom mapping, bin 0.15: disconnection mapping based tractograms binarized at 0.15, BCB: disconnectome mapping based on tractograms obtained with BCBtoolkit and binarized at 50 % overlap.

Symptom/JHU atlas tract	VLSM	bin 0.15	BCB
Right hemiparesis – left CST	1 %	28 %	27 %
Left hemiparesis – right CST	6 %	8 %	10 %
*mean*	*3 %*	*18 %*	*19 %*

**Fig. 5:**
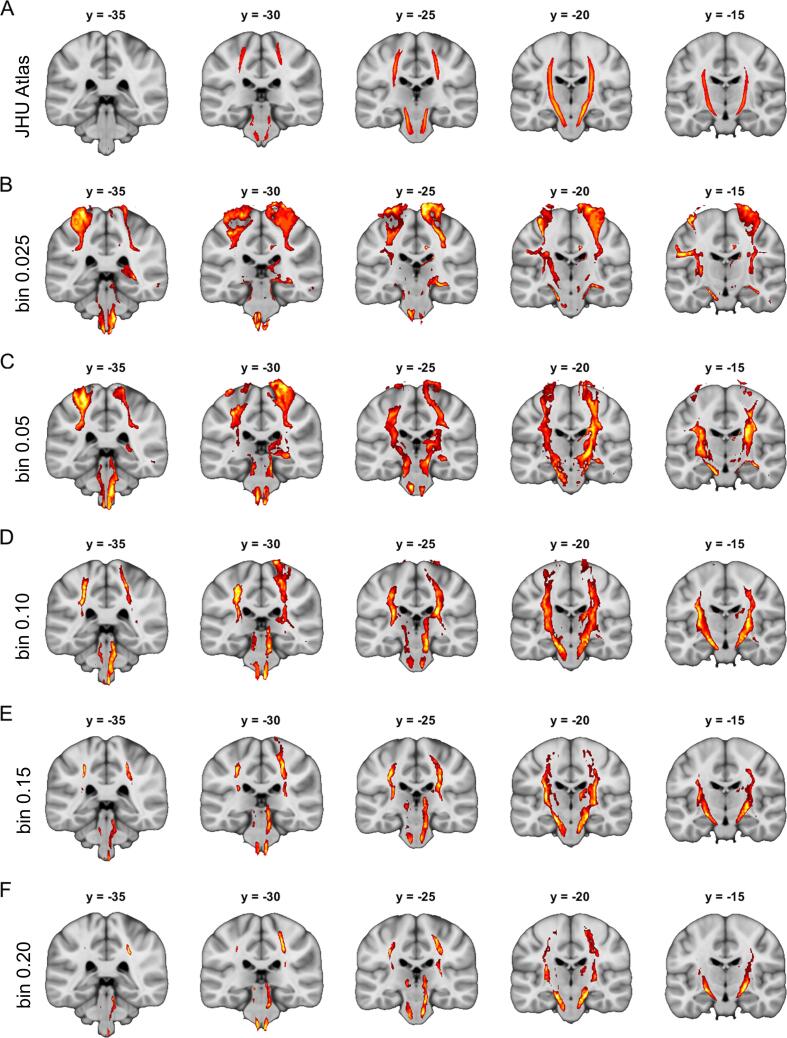
**Effect of binarizing threshold.** Panel A displays the left and right corticospinal tracts taken from the JHU white-matter tractography atlas (thresholded at 25 % overlap) which was used to simulate tract-specifics symptoms based on overlap with 70 real stroke lesions. The remaining rows show results from disconnection mapping analyses that relate maps binarized at (B) 0.025, (C) 0.05, (D) 0.10, (E) 0.15 and (F) 0.20 to the simulated symptom. All analyses in (B)–(F) were performed separately for the left and right corticospinal tract and then combined in one figure for display purposes. Inference is based on random permutation tests using Freedman-Lane procedure with 4,000 random permutations of the symptom label and lesion volume serving as a covariate of no interest restricted to regions with an overlap of at least 5 disconnection maps. All maps are thresholded at p(FWE) < 0.05 on the voxel-level. Coordinates refer to MNI space. Left hemisphere is displayed left.

First, we were interested in the optimal cutoff for binarization of disconnection maps obtained with our approach based on probabilistic tractography. Fig. 5 shows results from analyses comparing disconnection maps from patients with and without left or right CST damage binarized with five different cutoffs. As expected, we observed a tradeoff between sensitivity and specificity. Low cutoff values were associated with unacceptable low specificity but high sensitivity. Specificity increased with higher cutoff values. However, at high cutoff values, sensitivity began to drop. Based on all 14 tracts (Fig. 5 and SI Figs. 3–14), we found best balance between sensitivity and specificity at a cutoff value of 0.15. This is also evident in the Dice coefficients, which are highest (mean of 21 %) for this cutoff value (see Fig. 4). We therefore decided to use this cutoff value for all subsequent analyses.

**Fig. 6:**
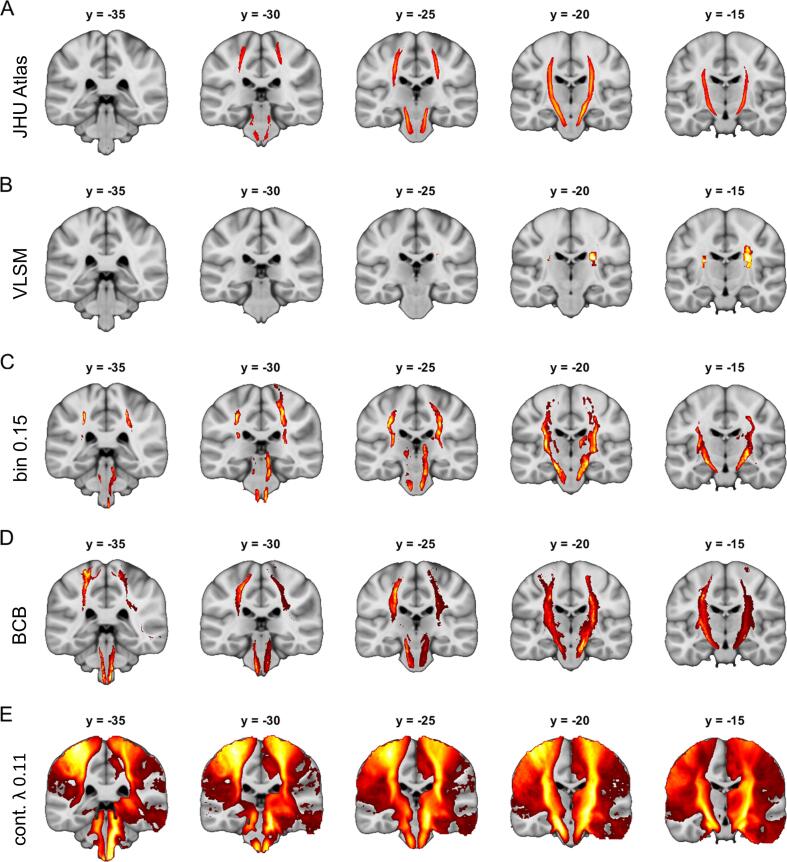
**Technical validation of structural disconnection mapping.** Panel A displays the left and right corticospinal tracts taken from the JHU white-matter tractography atlas (thresholded at 25 % overlap) which was used to simulate a tract-specific symptom based on overlap with individual 70 real stroke lesions. Panel B shows results from an ordinary VLSM analysis using the lesions and the simulated symptom. Panel C–E display results from different disconnection mapping analyses relating disconnection maps and the simulated symptom. Analyses were based on binarized disconnection maps generated using (C) our approach with a binarization threshold of 0.15, (D) BCBtoolkit binarized at ≥ 50 % tract overlap and (E) continuous disconnection maps with stabilized variance using power transformation (λ = 0.11). All analyses in (B)–(E) were performed separately for the left and right corticospinal tract and then combined in one figure for display purposes. Inference is based on random permutation tests using Freedman-Lane procedure with 4,000 random permutations of the symptom label and lesion volume serving as a covariate of no interest. Analyses in (B)–(D) are restricted to regions with an overlap of at least 5 lesions/disconnection maps. All maps are thresholded at p(FWE) < 0.05 on the voxel-level. Coordinates refer to MNI space. Left hemisphere is displayed left.

Second, we aimed to evaluate the performance of our disconnection mapping approach and the disconnectome mapping approach implemented in BCBtoolkit (Fig. 6, SI Figures 15–26). VLSM analyses which have been conducted as a methodological baseline in most cases either produced no significant result or patterns which did not overlap with the tract used to simulate the symptom (Panel B of Fig. 6 and SI Figures 15–26). Corresponding Dice coefficients were low with an average of only 3 %. However, both disconnection mapping approaches were able to depict the expected tracts for many tract-specific simulated symptoms (Panels C–D of Fig. 6 and SI Figure 15–26). Our method based on binarized disconnection maps reached a mean Dice coefficient of 21 % and BCBtoolkit even 31 %. This difference between our approach and BCBtoolkit was significant (p < 0.001, paired *t*-test). Although the approach to stabilize variance using power transformation correctly identified most tracts, it was associated with extremely poor overall specificity in most cases (Panel E of Fig. 6 and SI Figures 15–26) which is also mirrored in low Dice coefficients (average 7 %). We therefore refrained from further analyses using this approach.

### 3.2 Analysis 2 – Clinical validation

We applied voxelwise disconnection mapping to data from 316 stroke patients with regard to the symptom of hemiparesis. For this we used both, our own approach with a binarization threshold of 0.15 and the implementation in BCBtoolkit, separately for the symptom of right (71/316 patients) and left (61/316 patients) sided hemiparesis. VLSM analyses were performed for reference.

**Fig. 7:**
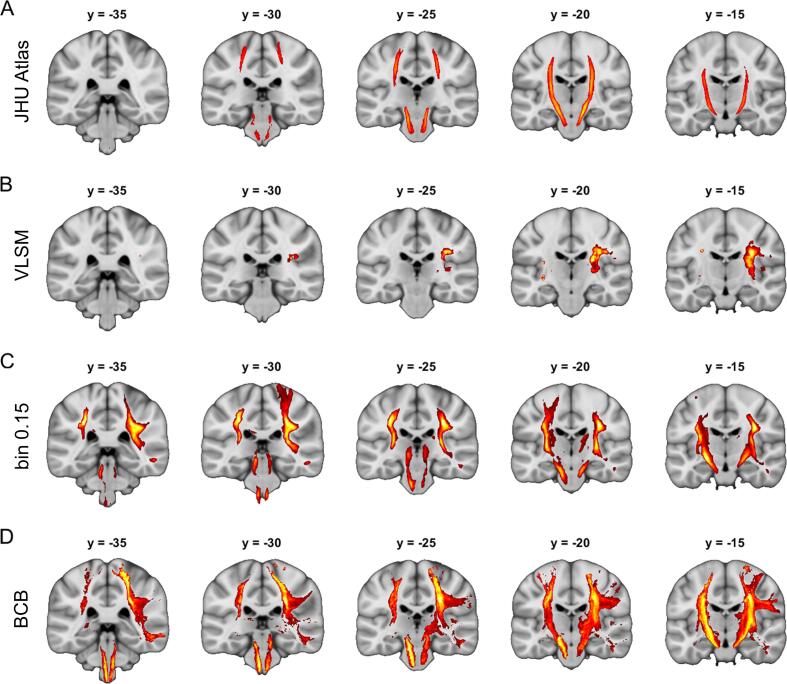
**Clinical validation of disconnection mapping.** Panel A displays the right and left corticospinal tract taken from the JHU white-matter tractography atlas (thresholded at 25 % overlap). Panel B shows VLSM analyses based on data of 316 patients with regard to the presence of a left or right sided hemiparesis. The same data has been used to perform disconnection mapping with our approach (Panel C) and with BCBtoolkit (Panel D). Left and right sided hemiparesis were analyzed separately and the results were combined in one figure for display purposes. All analyses in (B)–(D) are based on random permutation tests using Freedman-Lane procedure with 4,000 random permutations of the symptom label and lesion volume serving as a covariate of no interest restricted to regions with an overlap of at least 5 lesions/disconnection maps. All maps are thresholded at p(FWE) < 0.05 on the voxel-level. Coordinates refer to MNI space. Left hemisphere is displayed left.

VLSM analyses were able to detect parts of the right CST, but with poor overall spatial accuracy. The left CST could not be reliably identified using VLSM (Fig. 7B). However, both voxelwise disconnection mapping approaches were able to demonstrate that the contralateral CST is associated with hemiparesis after stroke for both hemispheres (Fig. 7C and D). Associated Dice coefficents also confirm the superiority of voxelwise disconnection mapping over VLSM for the symptom of hemiparesis (Table 1). In contrast to the technical validation, there was no substantial difference between our approach and BCBtoolkit in the Dice coefficients.

## 4 Discussion

Mapping symptoms to lesion locations has been a key method in cognitive neuroscience for over a century. It greatly advanced over time from case studies based on post-mortem dissections to in vivo MRI imaging, voxel-based analysis and permutation testing. Recently, structural connectome data has been utilized to leverage lesion-symptom mapping in situations where symptoms result from white-matter disconnection and where standard lesion-symptom mapping approaches might be less powerful. To this end, voxelwise disconnection mapping has been increasingly used in the recent years. With this study we aimed to address potential methodological constraints with a modified implementation of the approach. We further technically and clinically validated both, our and the prior implementation within BCBtoolkit based on simulated and real data in comparison to standard VLSM.

We found that standard VLSM analyses performed poorly in samples with underlying disconnection mechanisms. Most tracts in both analyses could not be identified with VLSM and the overall similarity between the desired tracts and the VLSM results was low. This failure is likely caused by the fact that widespread, non-overlapping lesions along a tract can cause the same symptom violating core assumptions of VLSM. Additionally, insufficient lesion overlap in the relevant regions also contributes, as many tracts do not overlap at all with the regions eligible for VLSM (Fig. 2B). It might, however, be speculated that (much) larger sample sizes could resolve this problem for VLSM. The disconnection mapping approach substantially extends the area of statistical inference. This might be an important reason for superiority of this approach compared to VLSM especially in moderately sized patient cohorts. For the same data and sample size, we were able to identify most of the analyzed tracts in patients with simulated symptoms with a clear superiority of BCBtoolkit over our own approach. Moreover, we were also able to identify the CST to be associated with contralateral hemiparesis after stroke based on real data from 316 patients. Both, our approach as well as BCBtoolkit performed equally well in these clinical validation analyses. In sum, we demonstrated that voxelwise disconnection mapping is a technically and clinically valid method to identify white-matter tracts whose damage is associated with certain symptoms and that it outperforms VLSM under these circumstances.

Based on Dice coefficients, disconnectome mapping as implemented in BCBtoolkit performed better than our approach in simulated symptoms but there was no clear difference between the two approaches for real hemiparesis data. However, since our approach based on probabilistic tractography is computationally more costly on the scale of magnitudes and BCBtoolkit is readily available for download and already includes the required connectome data, we recommend the use of BCBtoolkit in future disconnection mapping studies.

We found that the validity of disconnection mapping critically depends on methodological decisions which highlights the value of our study in retrospect and underlines the importance of validating novel methodological approaches. Based on our data obtained with different approaches and parameters, we would like to elaborate on several methodological details and make recommendations for best practice where appropriate.

### 4.1 Normative cohort sample size

We based our analysis on a normative cohort of 25 healthy subjects to perform fiber tracking. This number was sufficient to explain over 90 % of variance in the tractograms in comparison with a cohort of 187 subjects (SI Fig. 1A). Foulon and colleagues (2018) based their analysis on a sample of 10 healthy participants which was sufficient to explain over 70 % of variance (which is also in line with our data). As our analyses of disconnectome maps obtained with BCBtoolkit based on 10 healthy subjects produced valid results, we recommend a sample size of at least 10, ideally 25 healthy subjects to characterize the structural connectome. Since computational cost (especially relevant for probabilistic tractography) scales linearly with sample size, but an increase in explained variance per participant diminishes with increasing sample sizes, there might be only little benefit beyond sample sizes of 25 (SI Fig1A).

### 4.2 Thresholding disconnectome maps

In BCBtoolkit, maps are binarized on the individual level and subsequently summed up leading to an overlap of the disconnectome maps with values between 0 % and 100 %. Thresholding these disconnectome maps (i.e. keeping values ≥ 50 % and discarding values < 50 %) as performed in several prior studies (Alves et al., 2021, Foulon et al., 2018, Wiesen et al., 2020) and enabled by default in BCBtoolkit leads to significantly reduced variance for disconnection probabilities < 50 %. This leads to heteroscedasticity in the modelling errors in general linear models and is associated with inflated FWE rates in permutation testing (Huang et al., 2006). We therefore recommend binarizing of disconnectome maps prior to permutation testing instead of thresholding. It might be noted though, that also the binarized as well as the raw disconnectome maps (due to their binominal nature) are associated with some amounts of heteroscedasticity in the modelling errors. This issue could be solved in future studies by defining groups of exchangeability and performing random sign flipping instead of permuting the whole design matrix (Winkler et al., 2014).

### 4.3 Binarizing threshold

We found, that binarizing thresholds substantially influence the validity of the results. Low thresholds were associated with poor specificity and false positive results. This can be explained by poor spatial accuracy and overestimated tract size with lower thresholds. For our own approach based on probabilistic tractography with FSL, we found a threshold of 0.15 to be optimal. This threshold can, however, only be applied if tractography is performed with the same parameters (described in the methods section) as in the current analysis. Additionally, this threshold most likely also depends on the normative data itself and might be different for diffusion data obtained with different MR-scanners and/or with different scanning protocols.

For BCBtoolkit, we found that the threshold of ≥ 50 % overlap, which was motivated from the prior literature, led to valid results in the analyses presented in the paper. Nevertheless, we performed supplementary analyses to explore how different binarizing thresholds change results based on disconnectome maps obtained with BCBtoolkit. We repeated the technical validation analyses using disconnectome maps binarized at varying thresholds of 10 %, 20 %, 30 %, … and 100 % (see SI Table 2 and SI Figure 27). We found highest Dice coefficients (mean of 33 %) for binarizing thresholds of ≥ 60 % and ≥ 70 % overlap. We also repeated the clinical validation analyses using thresholds of 50 %, 60 % and 70 % (SI Table 3 and SI Figure 28). Here, we also found a slight superiority of these higher thresholds. We therefore recommend using thresholds of 60 % or 70 % in future analyses using BCBtoolkit. This recommendation refers to the data included in BCBtoolkit (n = 10) but might not be applicable for connectome data from other sources.

### 4.4 Power transformation

Raw disconnection maps are highly skewed towards small values (SI Fig. 2A). In order to preserve variance contained in raw disconnections maps though, we initially aimed to stabilize variance using power transformation (Box and Cox, 1964). We were able to accomplish stable variance in the data (SI Fig. 2B). However, results from the second level analyses displayed unacceptable low specificity. Supposedly, power transformation enhances spatial features in the disconnection maps which are only vaguely associated with the actual damaged tracts. This leads to poor spatial accuracy and indicates associations often in large areas around the actual tract (e.g. Fig. 6E) or even almost the whole cerebral hemisphere (SI Figure 23E). We therefore recommend refraining from using power transformations for variance stabilization in voxelwise disconnection mapping.

### 4.5 Binarizing individual tractograms

We found that binarizing individual tractograms in BCBtoolkit using a threshold of > 0 produced valid results on the group level. We therefore recommend using this threshold. However, a phantom study found (slightly) increased performance in deterministic tractography with more conservative thresholds (Sarwar et al., 2019). The exact influence of this threshold on disconnection mapping results remains elusive and might be investigated in future studies.

### 4.6 Limitations

The simulation of symptoms in the technical validation analyses relied on the arbitrarily defined threshold of 0.64 ml (= 4x4x4 voxels), i.e. patients with lesions overlapping > 0.64 ml with an atlas tract were defined as having a simulated symptom. To address the arbitrary nature of this threshold, we repeated the technical validation analysis using the disconnectome maps calculated with BCBtoolkit (binarized at ≥ 50 %). Symptoms were simulated based on thresholds of 0.16 ml, 0.64 ml and 2.56 ml overlap between individual lesion and atlas tracts (i.e. using a 4-fold smaller and larger threshold than in the initial analysis). There were small differences in the resulting statistical maps but Dice scores across all tracts were very similar (paired *t*-test between all pairs of analyses: p > 0.50, see SI Figure 29). Therefore, the results in the technical validation analyses seem not critically dependent on the exact choice of volumetric threshold for symptom simulation.

### 4.7 Conclusion

Voxelwise disconnection mapping is a novel method, which has been used increasingly in the recent years. We performed thorough validation of this method and discussed some of the methodological details. Disconnection mapping is a technically and clinically valid method to uncover neural substrates of symptoms caused by disconnection following focal brain lesions. Under these circumstances it is also superior to VLSM. We recommend the use of BCBtoolkit for disconnection mapping and binarizing the maps (with a cutoff of ≥ 50–70 %) when performing permutation tests. We also found that slight methodological changes can dramatically decrease the validity of this method which highlights the importance of validation studies for novel methods in the field of neuroimaging.

## Funding

Dorothee Saur and Julian Klingbeil (SA 1723/5-1) are supported by the Deutsche Forschungsgemeinschaft. Max Wawrzyniak is supported by the Clinician Scientist Program of the Medical Faculty of the University of Leipzig. Open access publication is supported by the University of Leipzig.

## CRediT authorship contribution statement

**Max Wawrzyniak:** Conceptualization, Formal analysis, Methodology, Software, Writing – original draft, Writing – review & editing. **Anika Stockert:** Writing – review & editing. **Julian Klingbeil:** Writing – review & editing. **Dorothee Saur:** Conceptualization, Writing – review & editing.

## Declaration of Competing Interest

The authors declare that they have no known competing financial interests or personal relationships that could have appeared to influence the work reported in this paper.

## Data Availability

See methods section.
