# Supplementary material for: Voxelwise structural disconnection mapping: Methodological validation and recommendations
Source: Neuroimage Clin. 2022 Jul 29;35:103132. doi: 10.1016/j.nicl.2022.103132 (PMC9421530; doi:10.1016/j.nicl.2022.103132)
Supplement: Supplementary Data 1 [file mmc1.pdf]

Supplementary Information

Voxelwise structural disconnection mapping: methodological validation and recommendations

Max Wawrzyniak, MD[1], Anika Stockert, MD[1], Julian Klingbeil, MD[1], Dorothee Saur, MD[1]

[1]Language and Aphasia Laboratory, Department of Neurology, University of Leipzig Medical Center, Leipzig, Germany

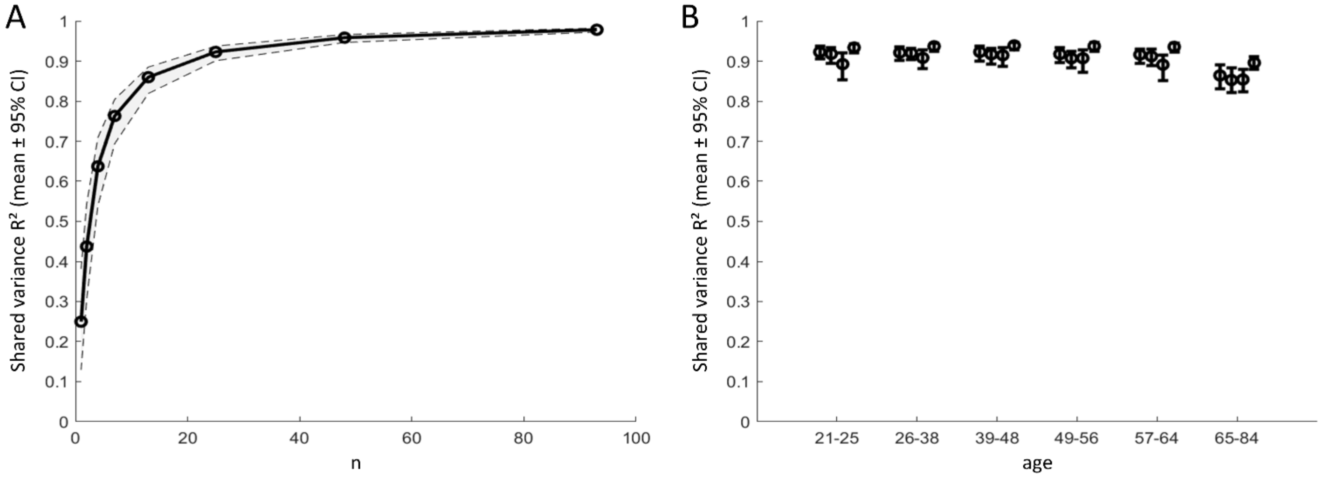

SI Figure 1. Influence of size and age of normative cohort. Panel A shows shared variance between mean connectivity of differently sized cohorts and all remaining subjects (∑ = 187) for a representative lesion. Panel B shows shared variance between random groups of 25 participants within different age groups and random participants irrespective of age for four random lesions. Both plots display shared variance (R^2) and it's 95% confidence interval based on 1,000 permutations.

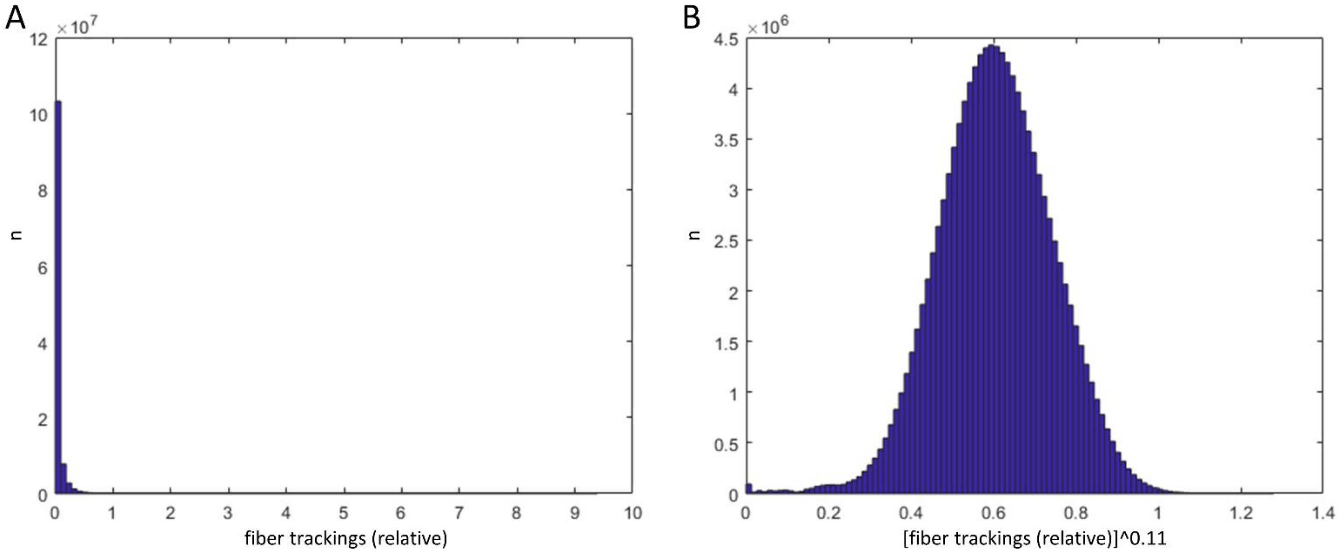

SI Figure 2. Distribution of raw and power transformed visitation maps. Panel A displays the distribution of values within raw disconnection maps aggregated across all 70 patients in the technical validation cohort. Panel B displays the distribution of the same values after power transformation with a parameter of 0.11 (i.e. $y = x^{0.11}$).

JHU atlas tract	VLSM	bin 0.025	bin 0.05	bin 0.1	bin 0.15	bin 0.2	BCB	cont. λ 0.11
Anterior thalamic radiation L	2%	2%	13%	32%	38%	43%	**52%**	9%
Anterior thalamic radiation R	0%	2%	7%	22%	**22%**	7%	21%	21%
Corticospinal tract L	10%	6%	19%	28%	26%	23%	**41%**	3%
Corticospinal tract R	19%	1%	10%	21%	30%	**35%**	32%	2%
Forceps major	0%	4%	16%	22%	18%	7%	**46%**	12%
Inferior fronto-occipital fasciculus L	1%	1%	4%	21%	27%	29%	**32%**	4%
Inferior fronto-occipital fasciculus R	0%	0%	1%	14%	22%	18%	**28%**	14%
Inferior longitudinal fasciculus L	0%	0%	4%	15%	14%	**24%**	16%	9%
Inferior longitudinal fasciculus R	0%	0%	0%	4%	9%	3%	**23%**	0%
Superior longitudinal fasciculus L	0%	4%	21%	31%	18%	4%	**39%**	11%
Superior longitudinal fasciculus R	7%	9%	16%	23%	27%	28%	**32%**	3%
Uncinate fasciculus L	0%	0%	1%	0%	3%	**8%**	5%	1%
Superior longitudinal fasciculus (temporal part) L	0%	0%	2%	32%	23%	9%	**40%**	7%
Superior longitudinal fasciculus (temporal part) R	5%	0%	0%	8%	16%	21%	**27%**	4%
mean	*3%*	*2%*	*8%*	*19%*	*21%*	*19%*	***31%***	*7%*

SI Table 1. Dice coefficients – technical validation. This table displays similarity between the atlas tract used to simulate the symptom and the results from the different analyses. Higher values imply higher similarity. Abbreviations: VLSM: voxel-based lesion-symptom mapping, bin 0.x: disconnection mapping based tractograms binarized at different thresholds, BCB: disconnection mapping based on tractograms obtained with BCBtoolkit and binarized at 50 % overlap, cont. λ 0.11: continuous disconnection maps which were power transformed with $\lambda = 0.11$.

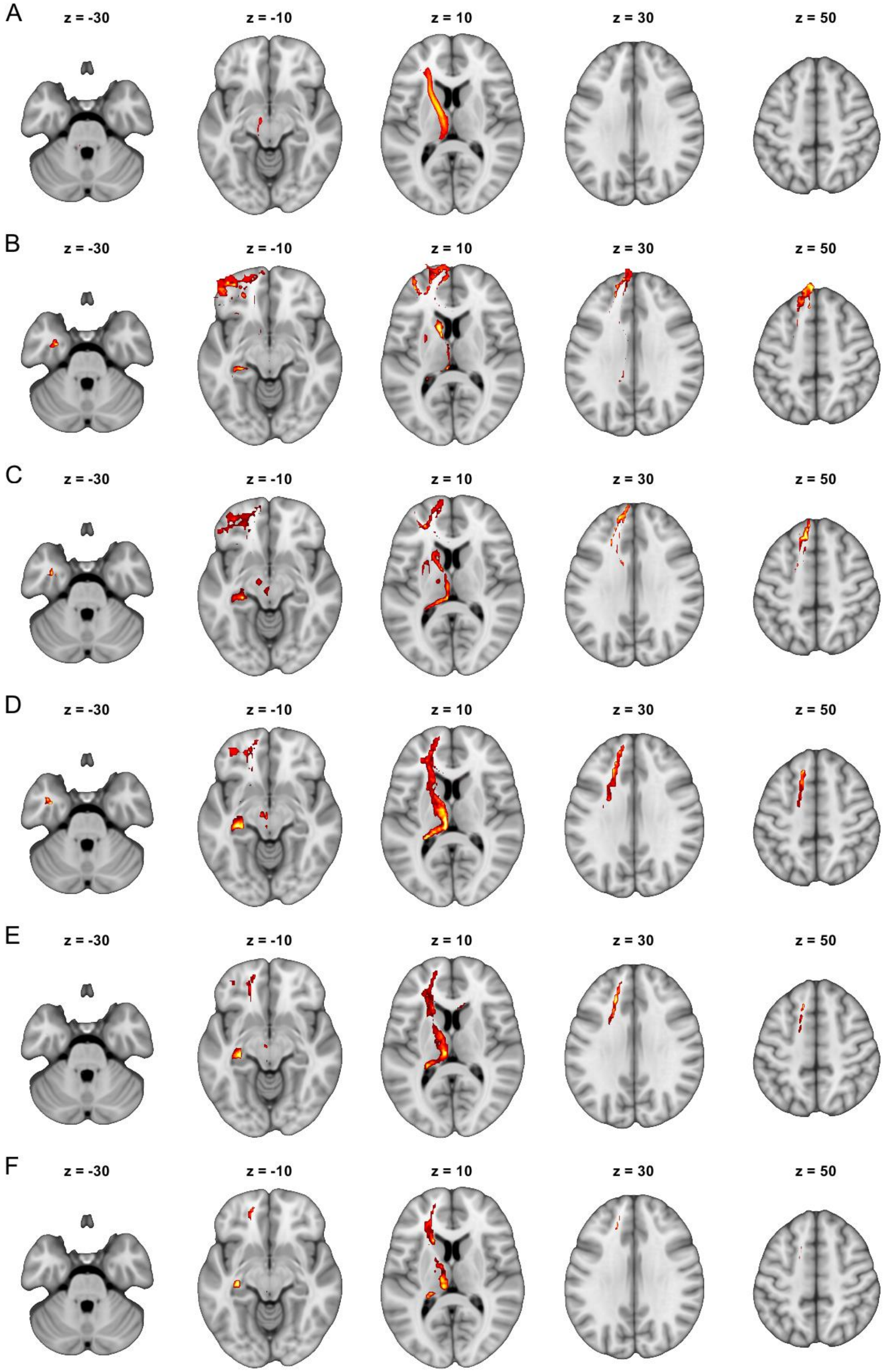

SI Figure 3. Influence of binarizing threshold for Anterior thalamic radiation L. Please consult legend of Figure 3 in the manuscript for explainations.

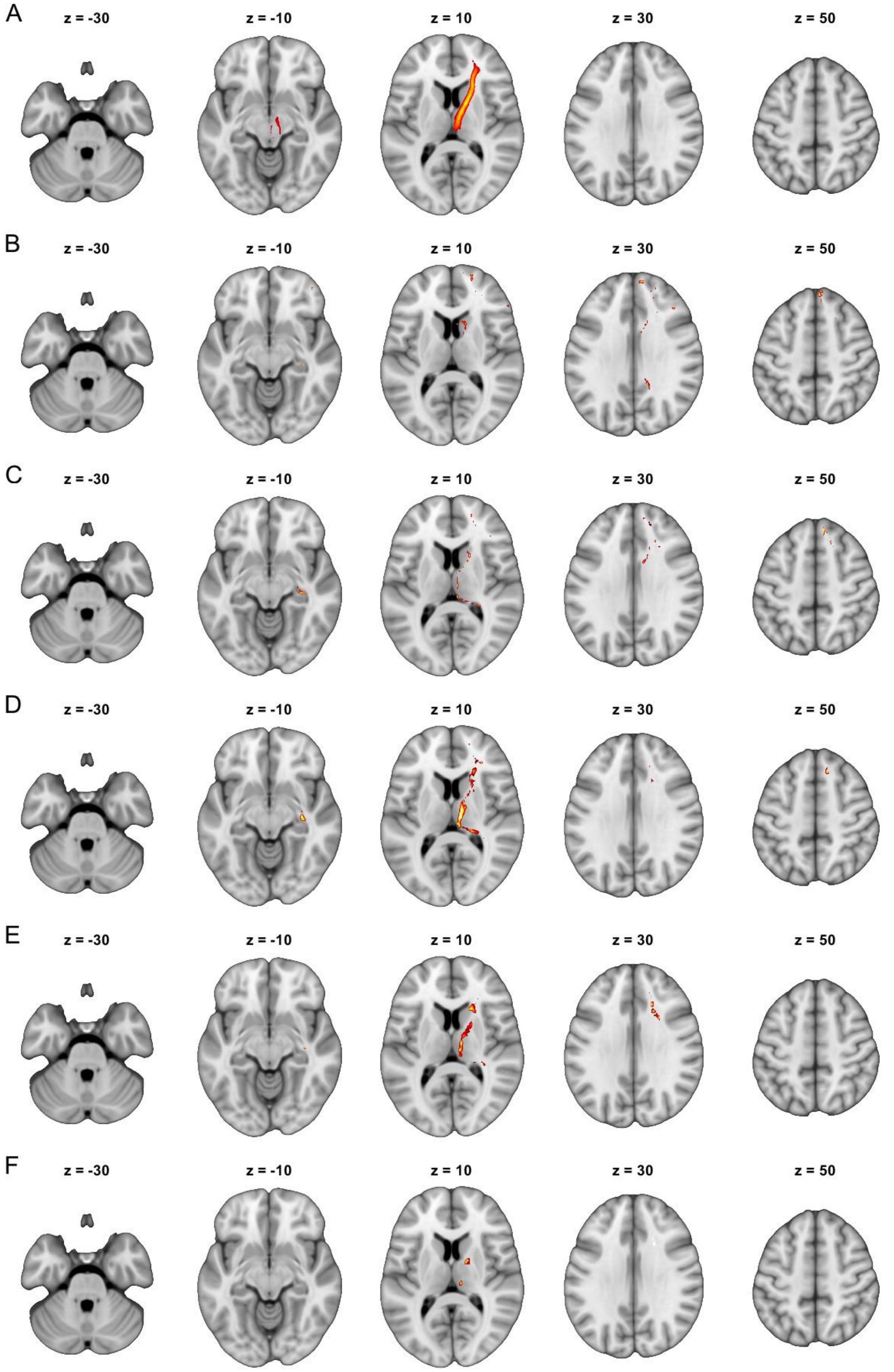

SI Figure 4. Influence of binarizing threshold for Anterior thalamic radiation R. Please consult legend of Figure 3 in the manuscript for explainations.

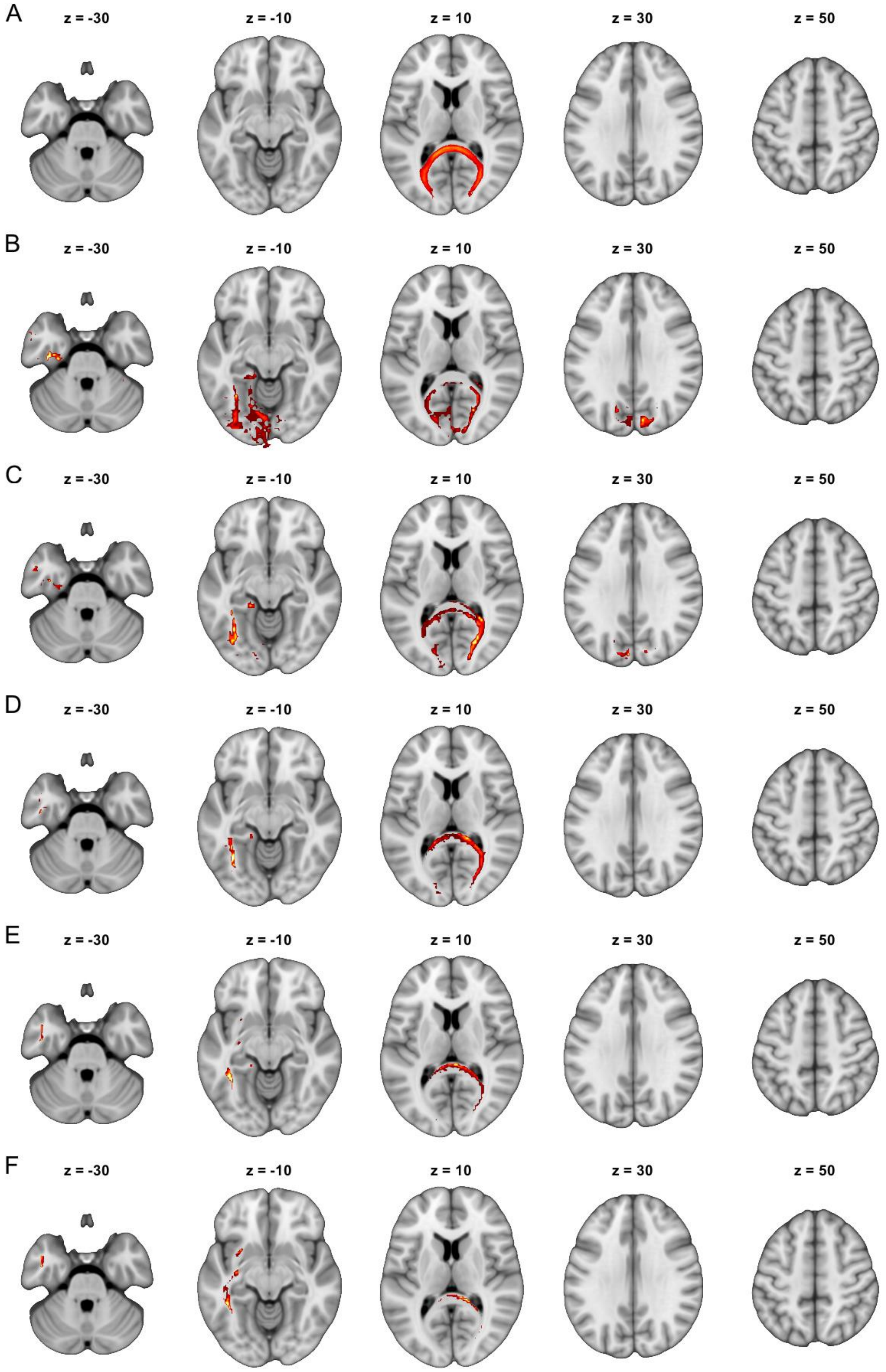

SI Figure 5. Influence of binarizing threshold for Forceps major. Please consult legend of Figure 3 in the manuscript for explainations.

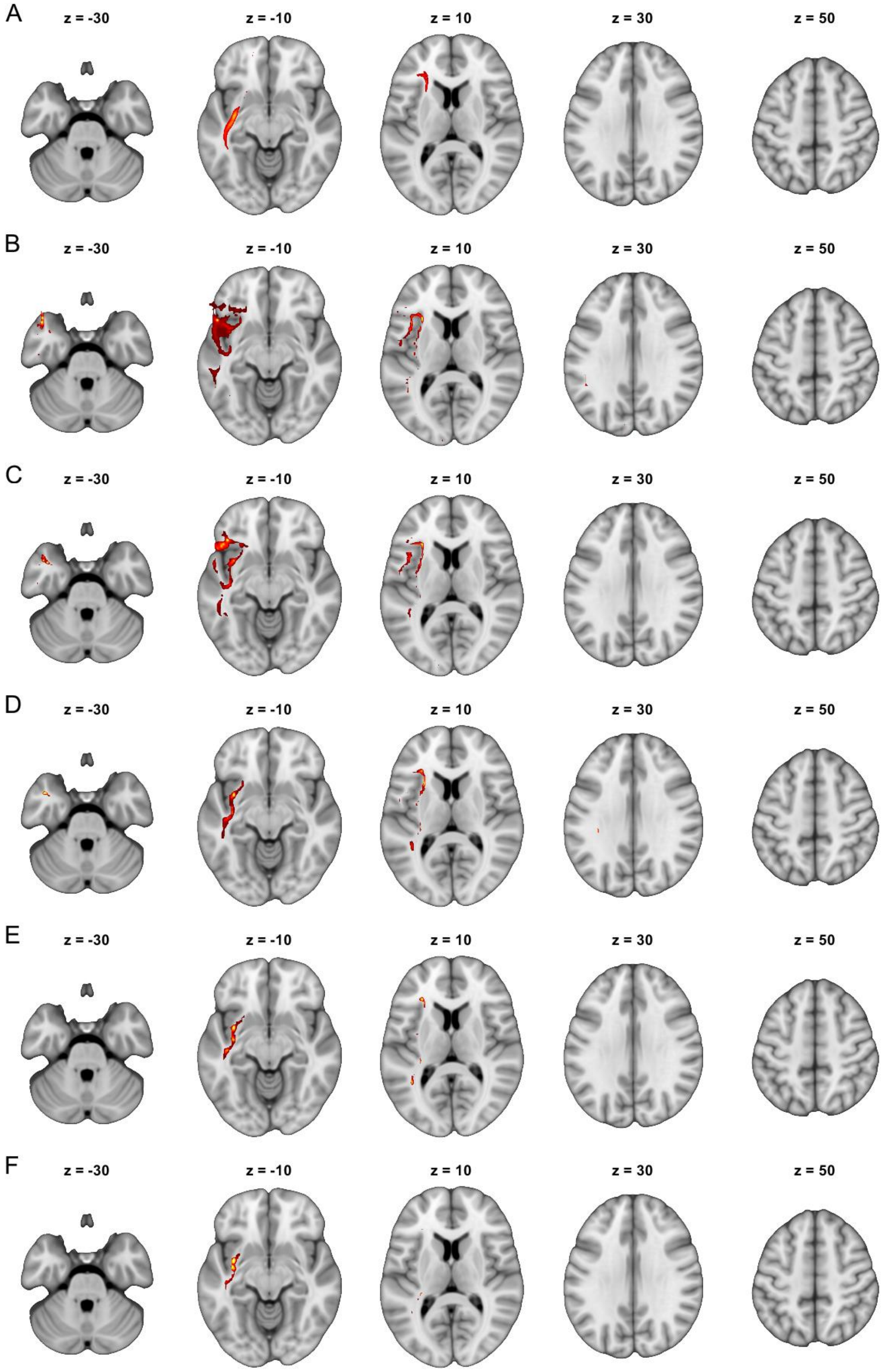

SI Figure 6. Influence of binarizing threshold for Inferior fronto-occipital fasciculus L. Please consult legend of Figure 3 in the manuscript for explainations.

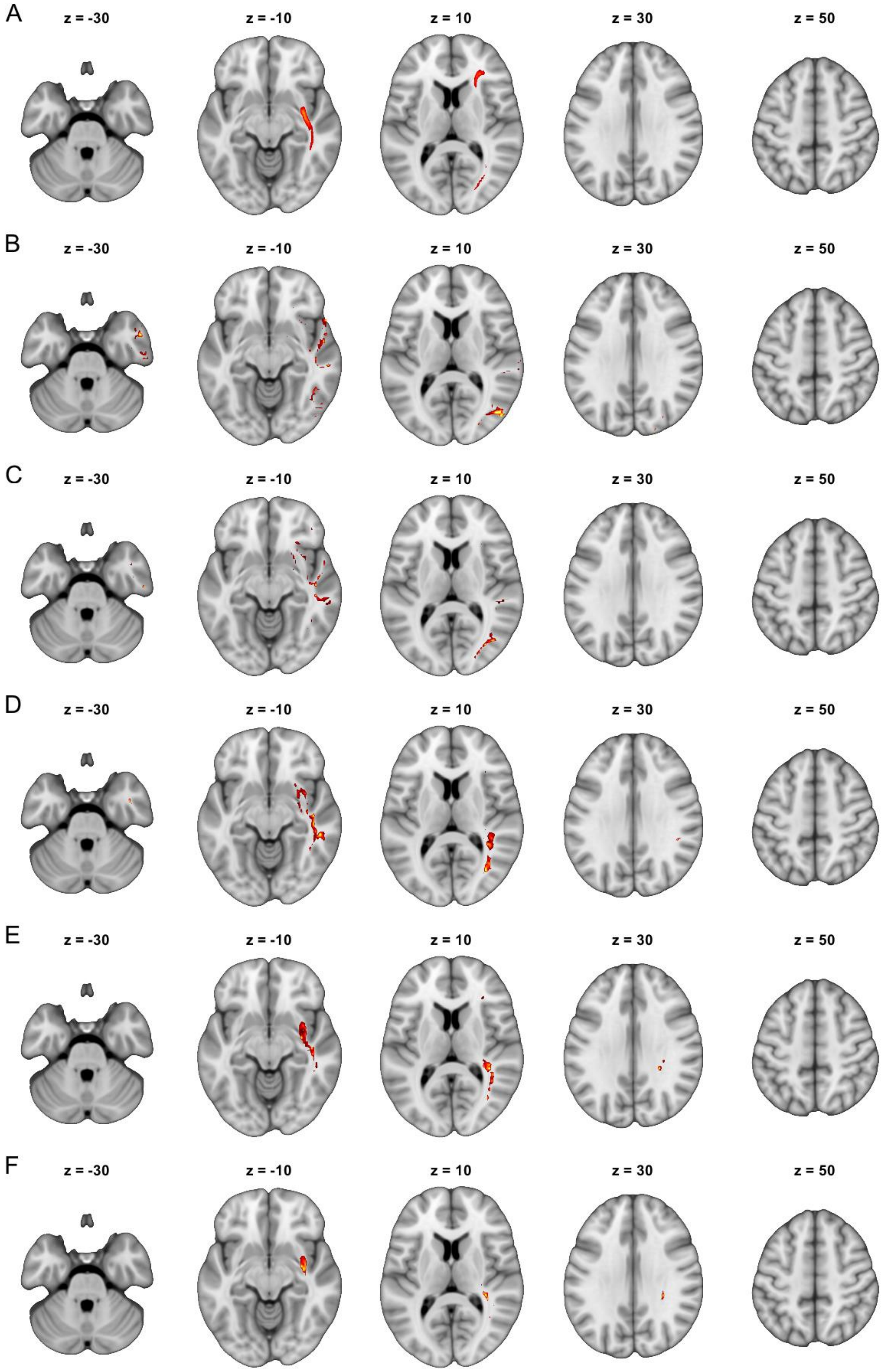

SI Figure 7. Influence of binarizing threshold for Inferior fronto-occipital fasciculus R. Please consult legend of Figure 3 in the manuscript for explainations.

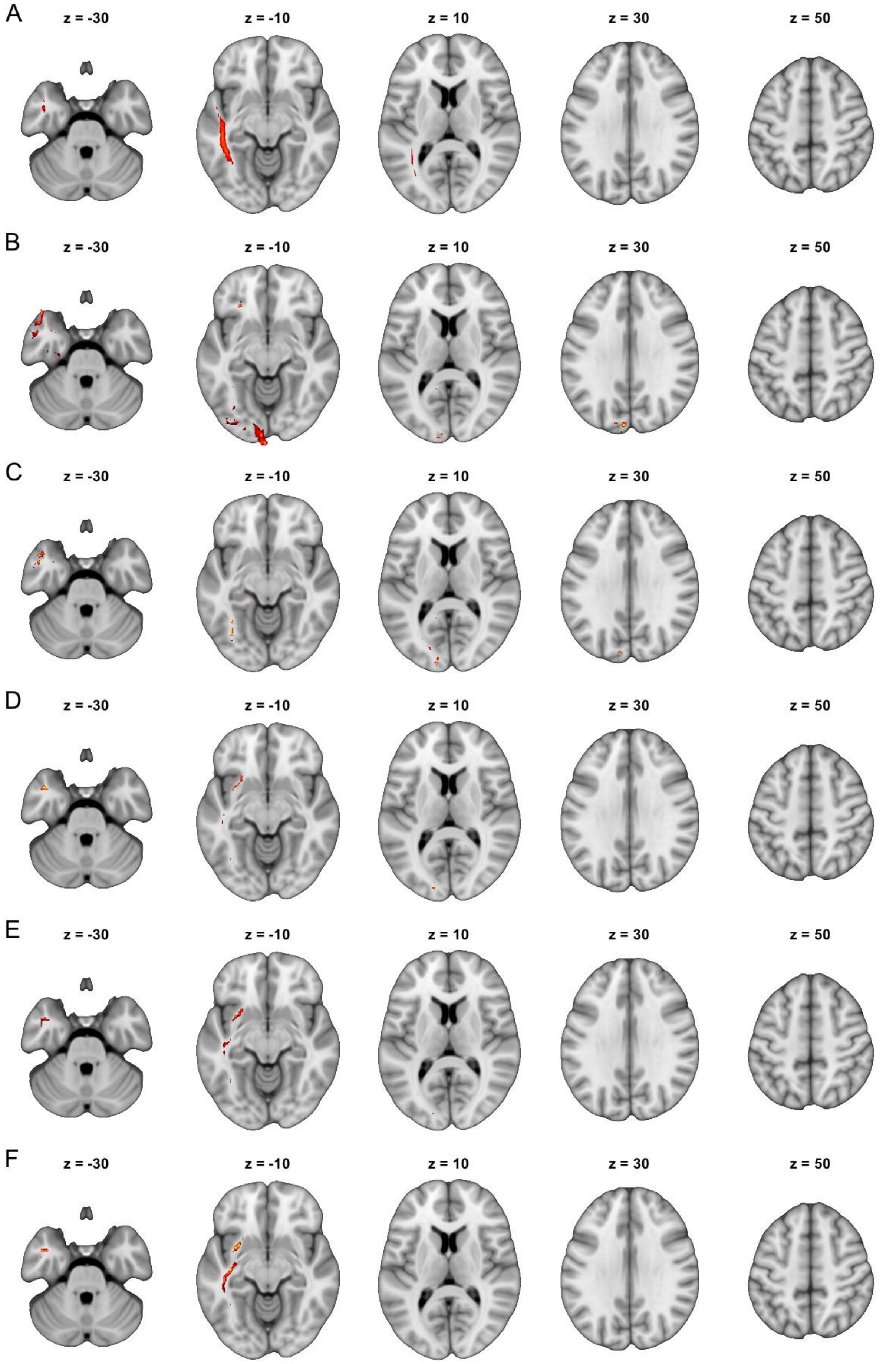

SI Figure 8. Influence of binarizing threshold for Inferior longitudinal fasciculus L. Please consult legend of Figure 3 in the manuscript for explainations.

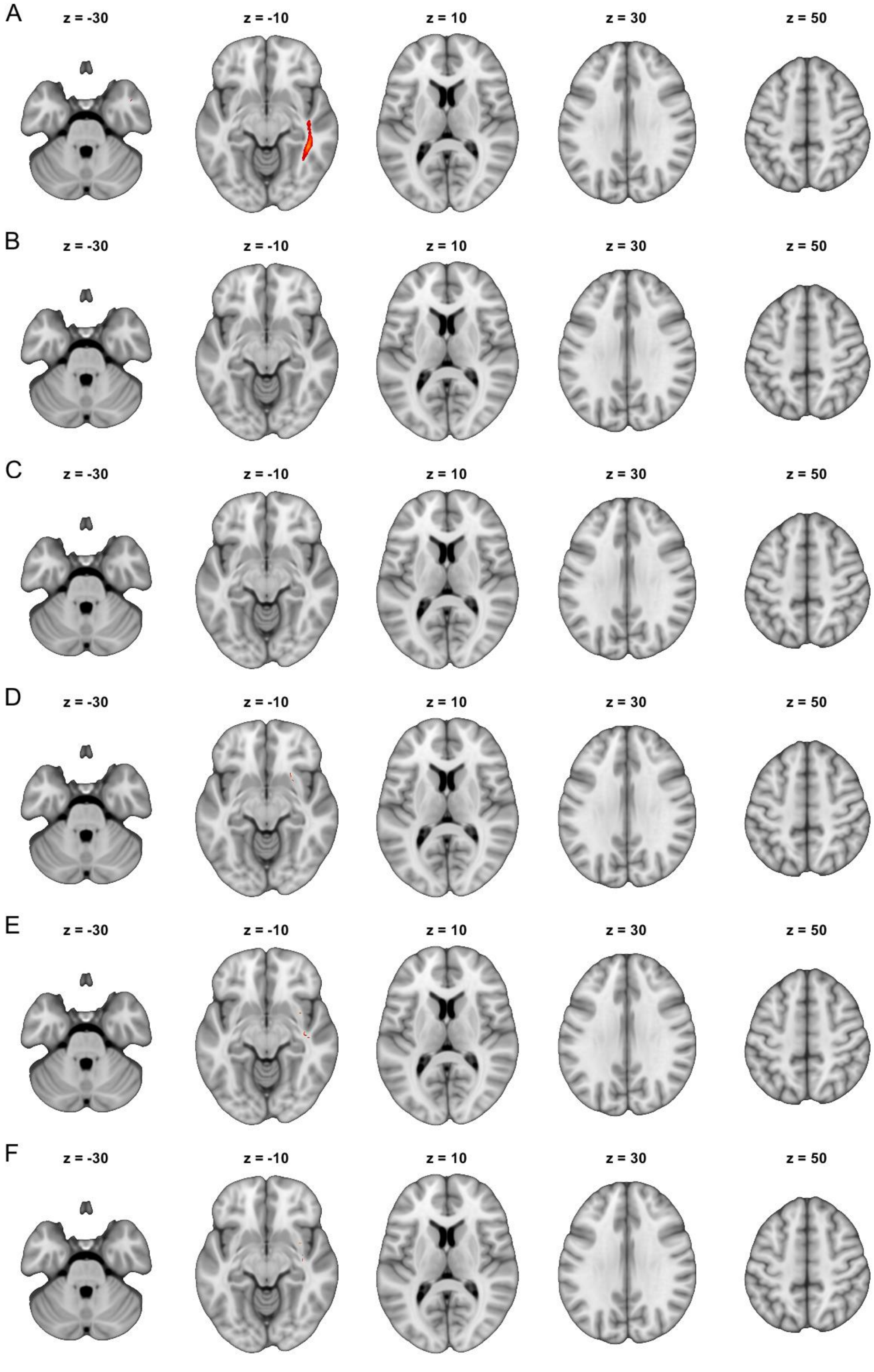

SI Figure 9. Influence of binarizing threshold for Inferior longitudinal fasciculus R. Please consult legend of Figure 3 in the manuscript for explainations.

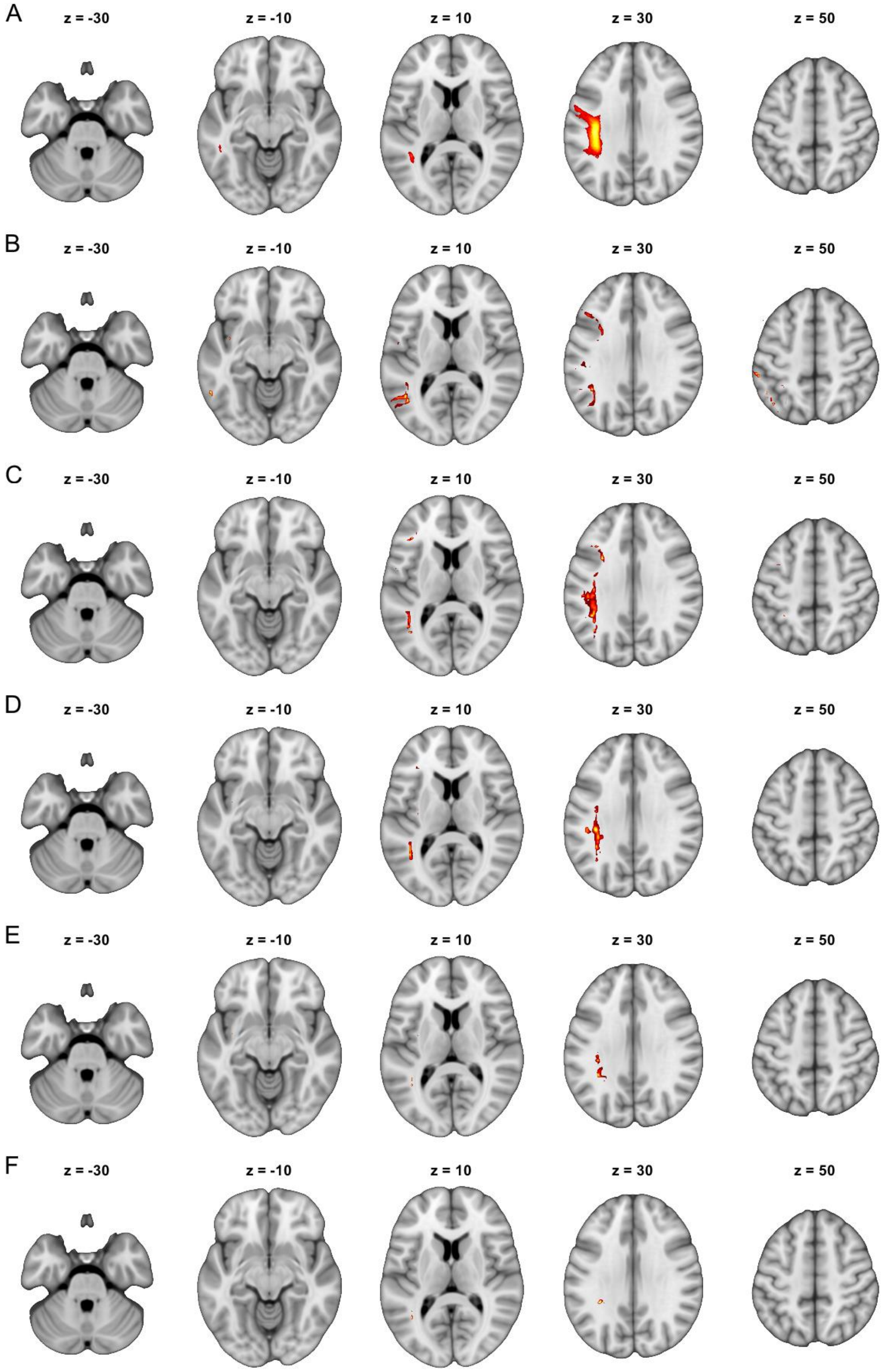

SI Figure 10. Influence of binarizing threshold for Superior longitudinal fasciculus L. Please consult legend of Figure 3 in the manuscript for explainations.

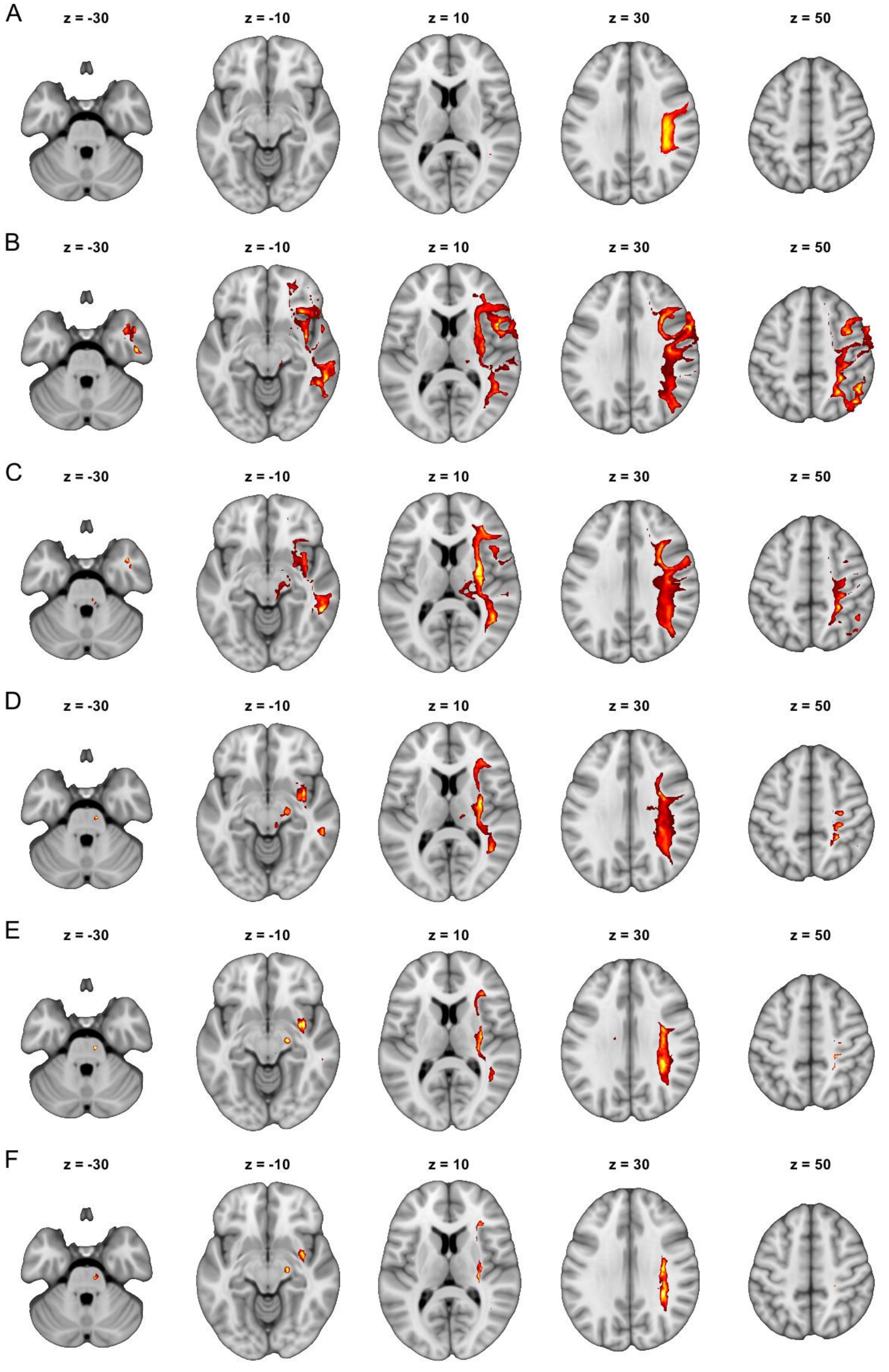

SI Figure 11. Influence of binarizing threshold for Superior longitudinal fasciculus R. Please consult legend of Figure 3 in the manuscript for explainations.

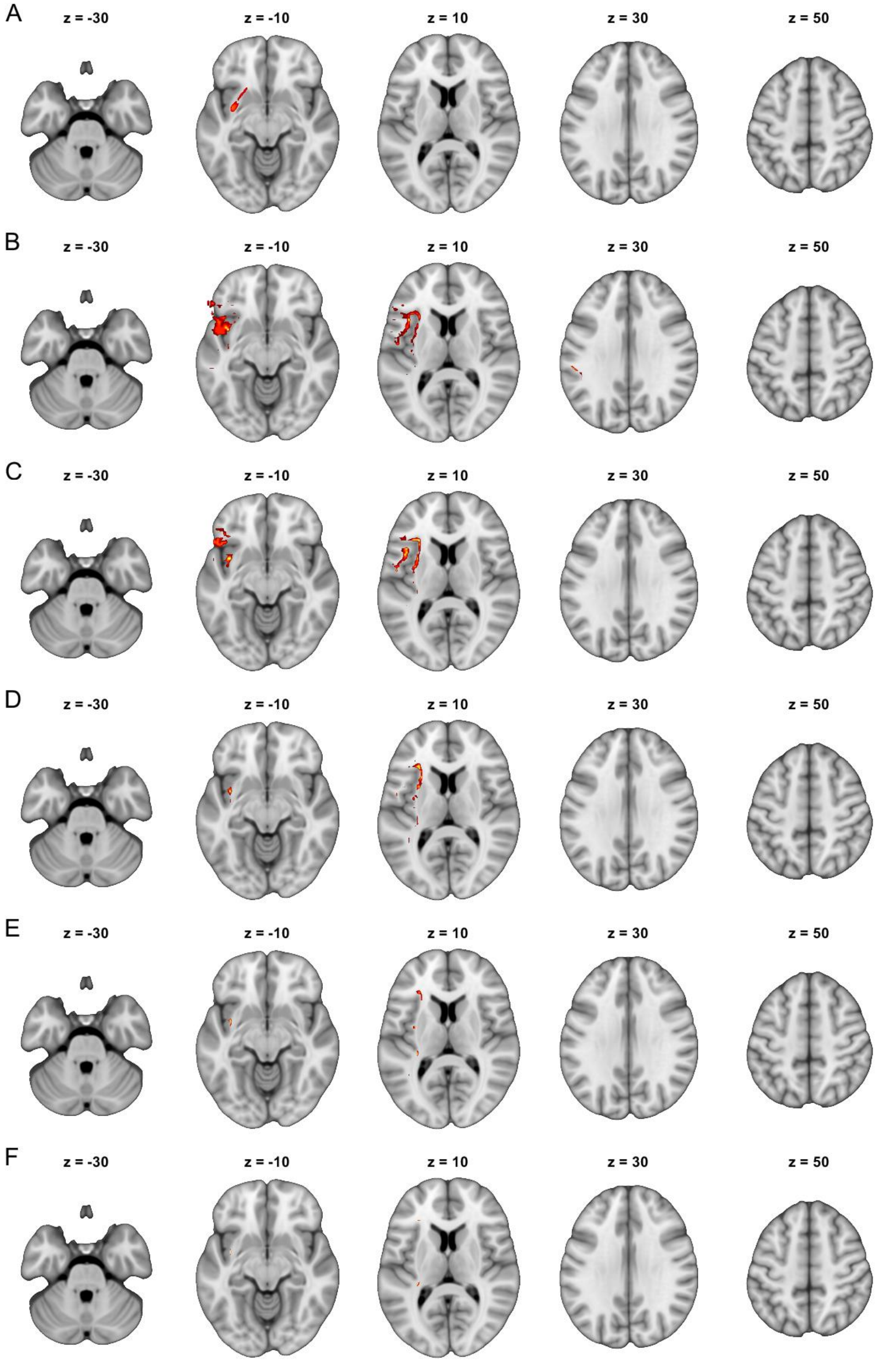

SI Figure 12. Influence of binarizing threshold for Uncinate fasciculus L. Please consult legend of Figure 3 in the manuscript for explainations.

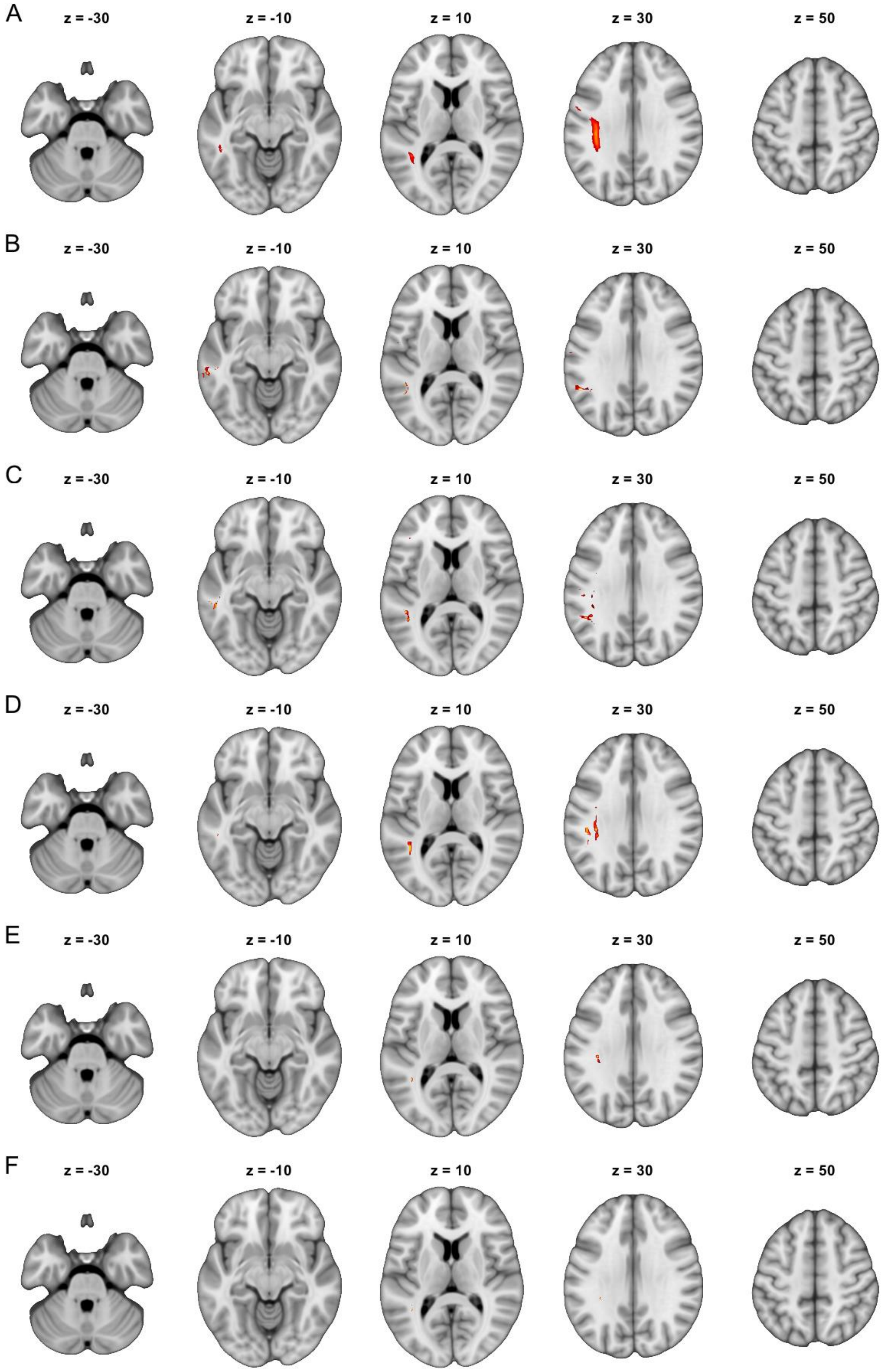

SI Figure 13. Influence of binarizing threshold for Superior longitudinal fasciculus (temporal part) L. Please consult legend of Figure 3 in the manuscript for explainations.

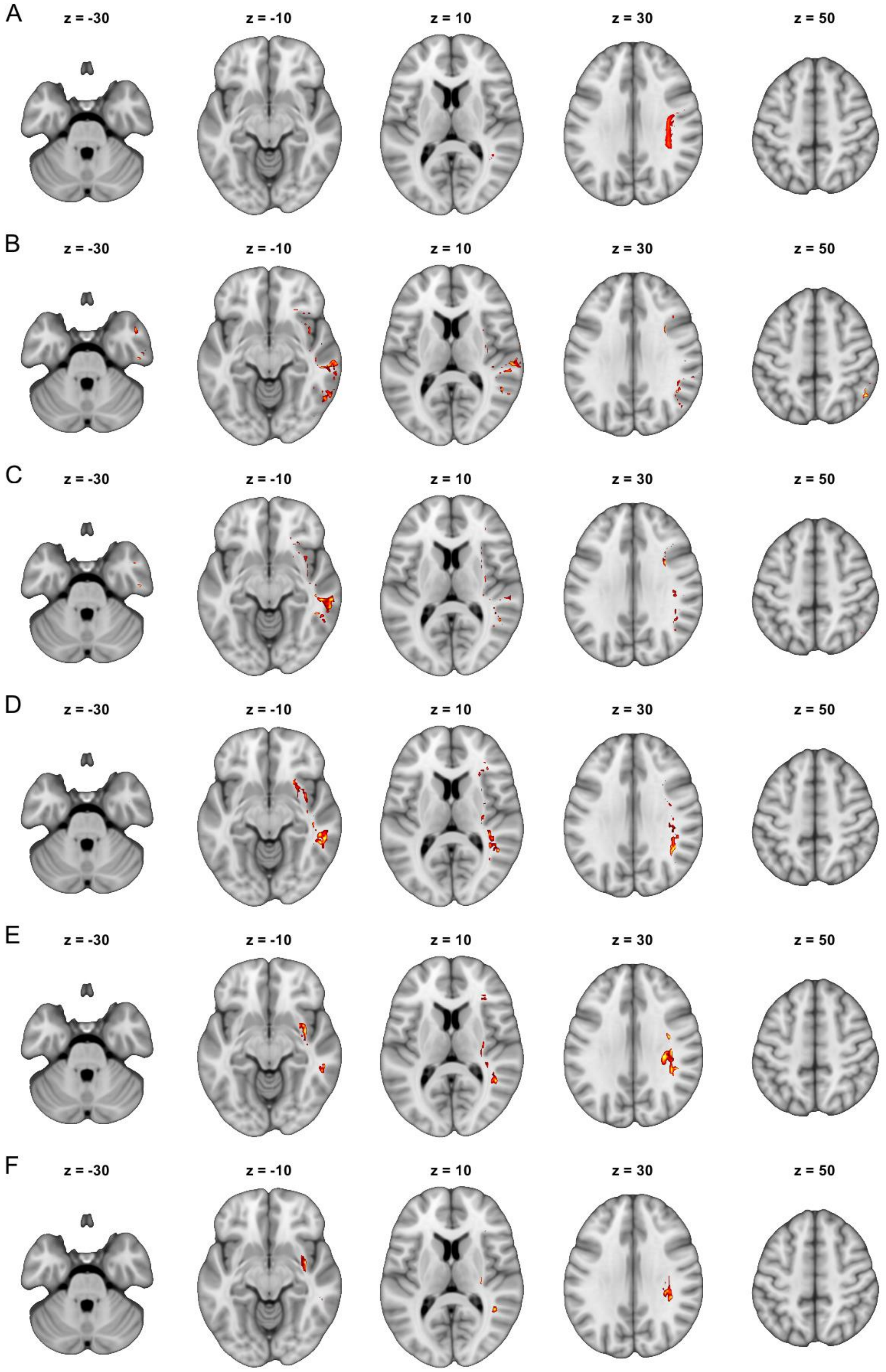

SI Figure 14. Influence of binarizing threshold for Superior longitudinal fasciculus (temporal part) R. Please consult legend of Figure 3 in the manuscript for explainations.

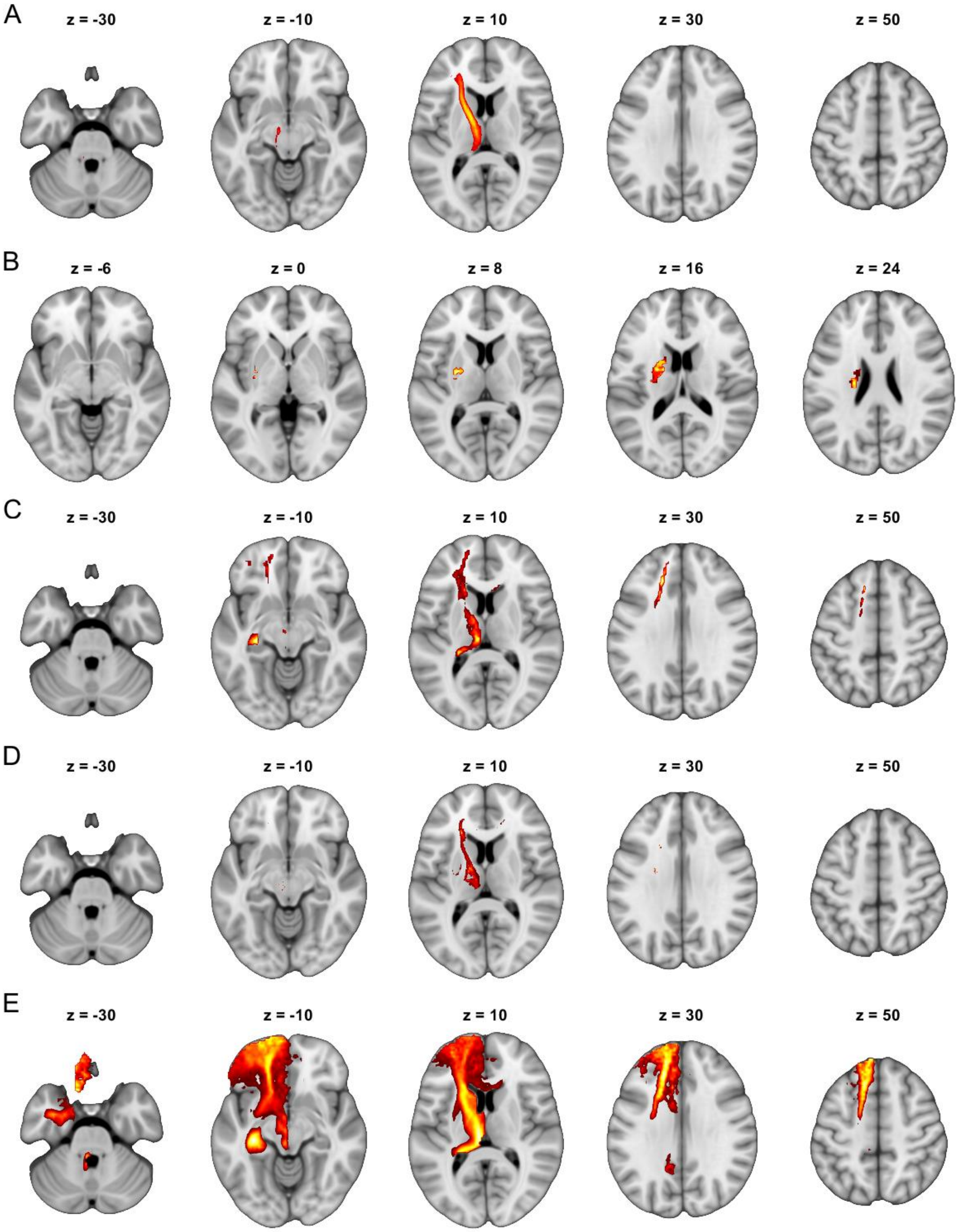

SI Figure 15. Technical validation of structural disconnection mapping for Anterior thalamic radiation L. Please consult legend of Figure 4 in the manuscript for explainations.

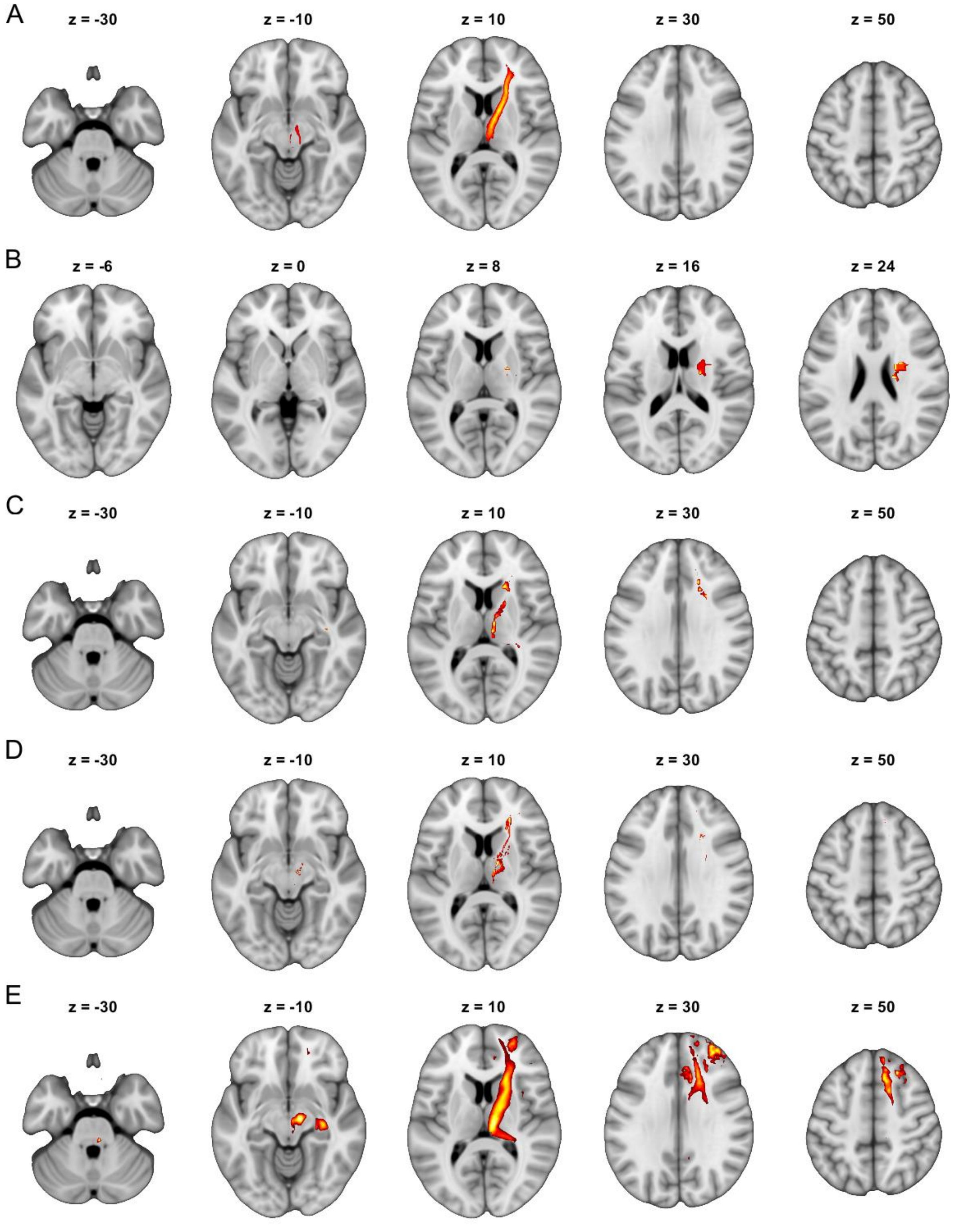

SI Figure 16. Technical validation of structural disconnection mapping for Anterior thalamic radiation R. Please consult legend of Figure 4 in the manuscript for explainations.

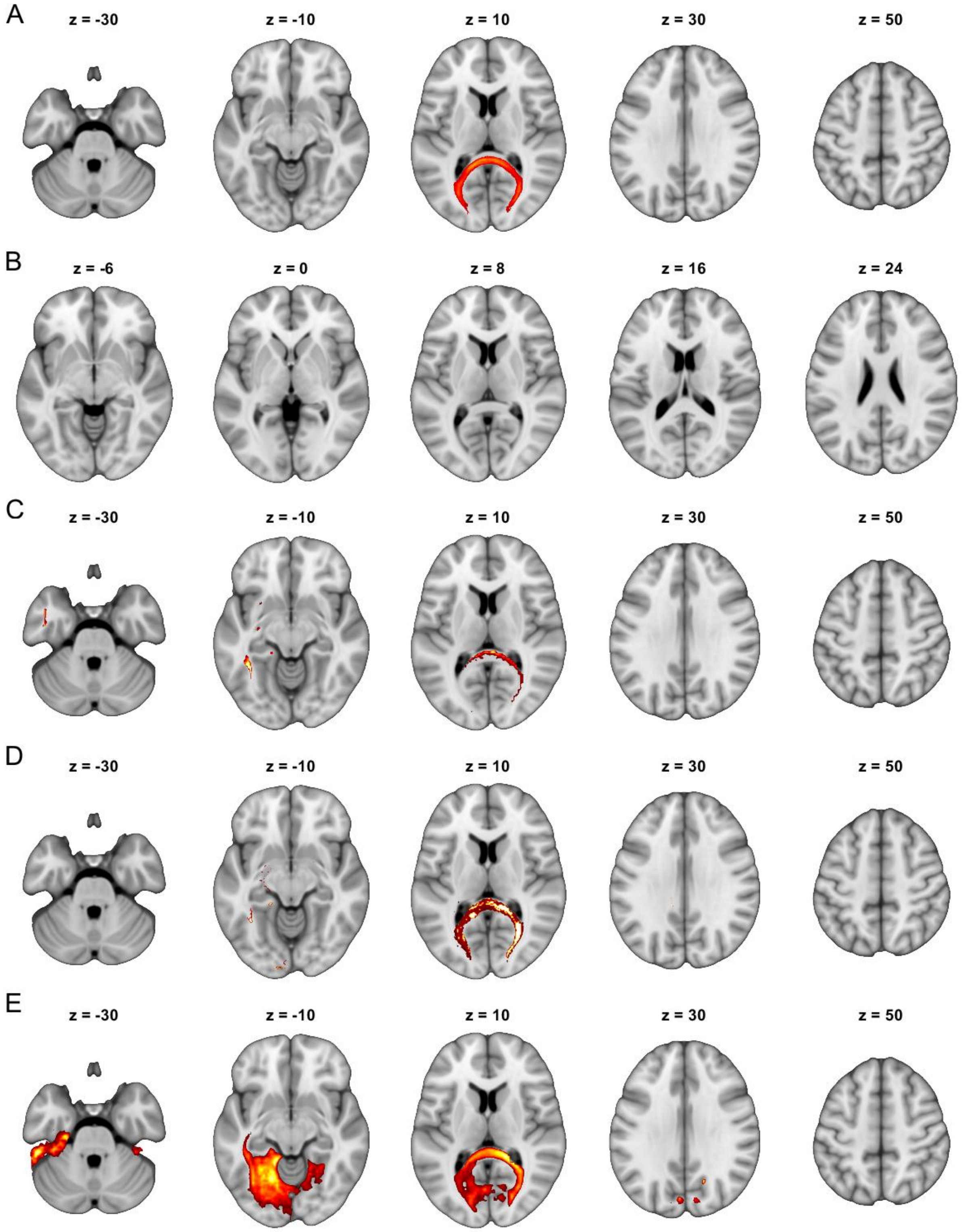

SI Figure 17. Technical validation of structural disconnection mapping for Forceps major. Please consult legend of Figure 4 in the manuscript for explainations.

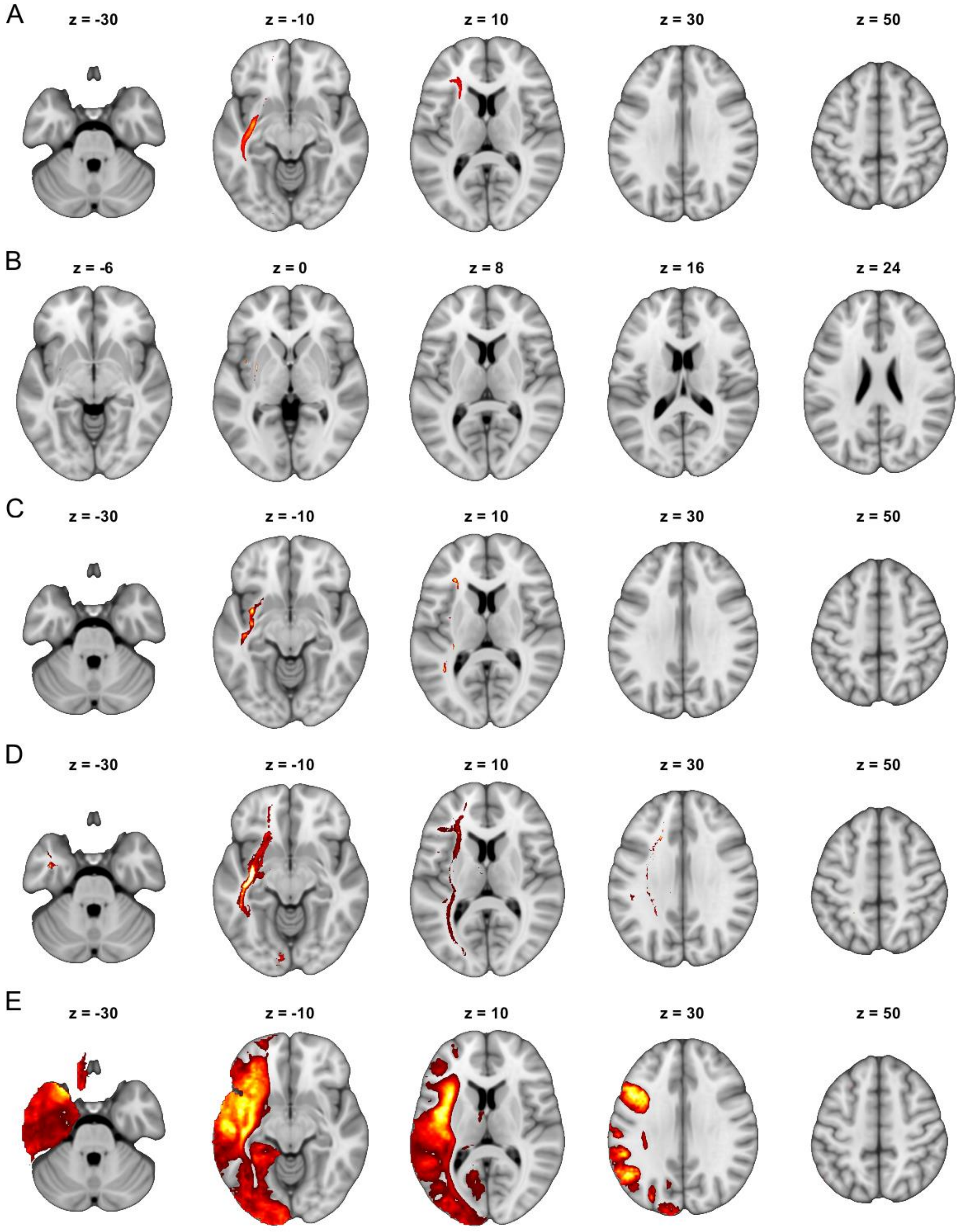

SI Figure 18. Technical validation of structural disconnection mapping for Inferior fronto-occipital fasciculus L. Please consult legend of Figure 4 in the manuscript for explainations.

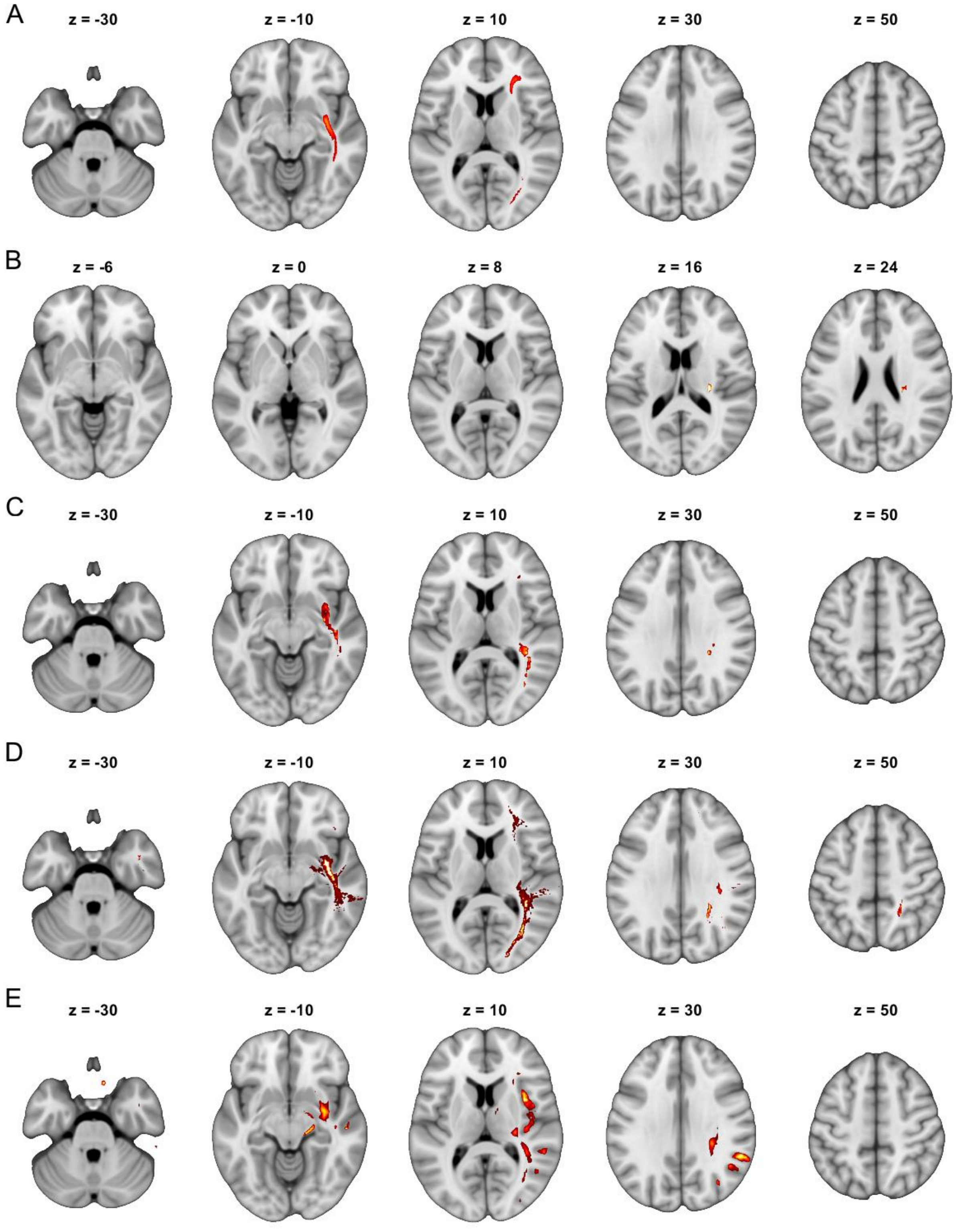

SI Figure 19. Technical validation of structural disconnection mapping for Inferior fronto-occipital fasciculus R. Please consult legend of Figure 4 in the manuscript for explainations.

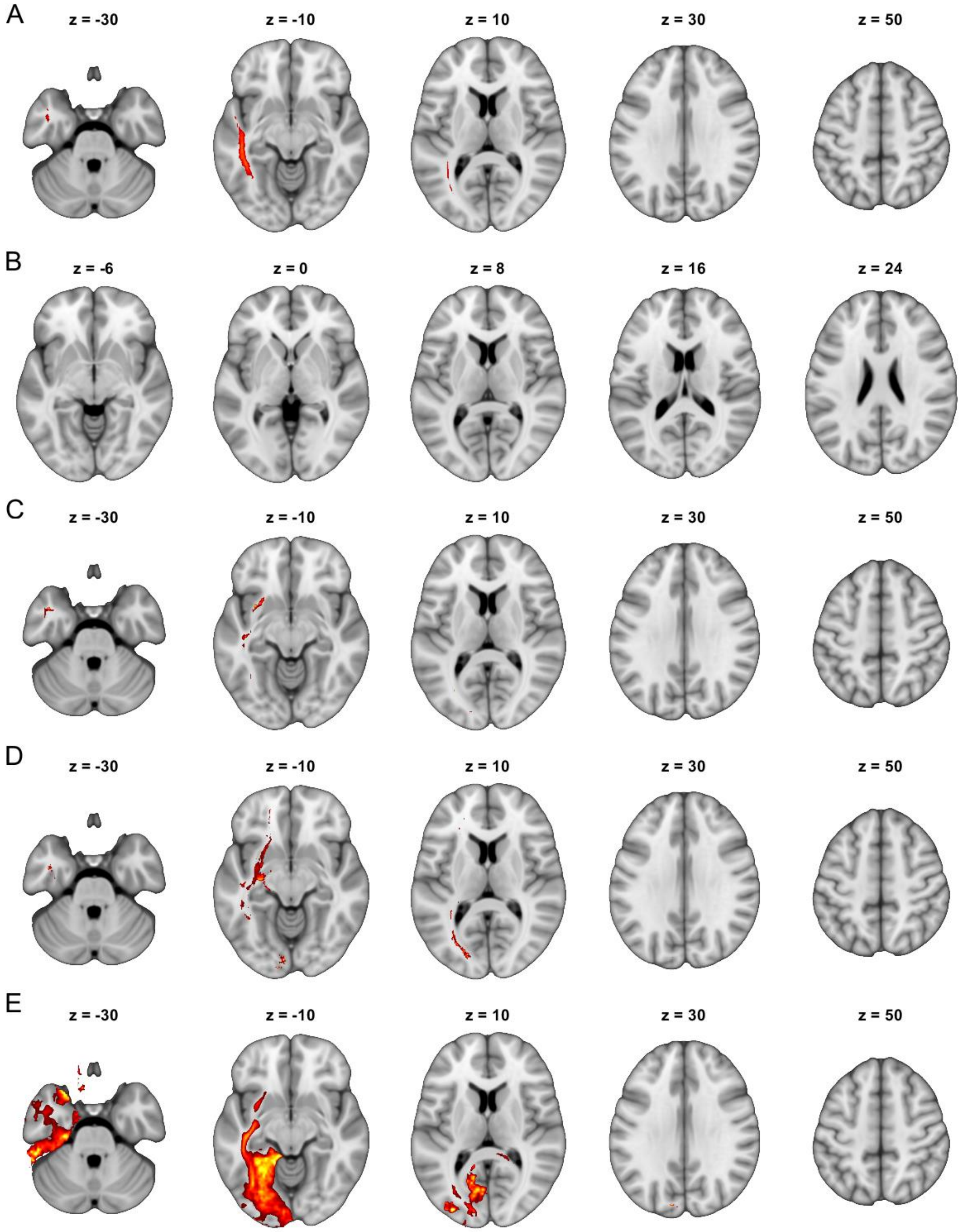

SI Figure 20. Technical validation of structural disconnection mapping for Inferior longitudinal fasciculus L. Please consult legend of Figure 4 in the manuscript for explainations.

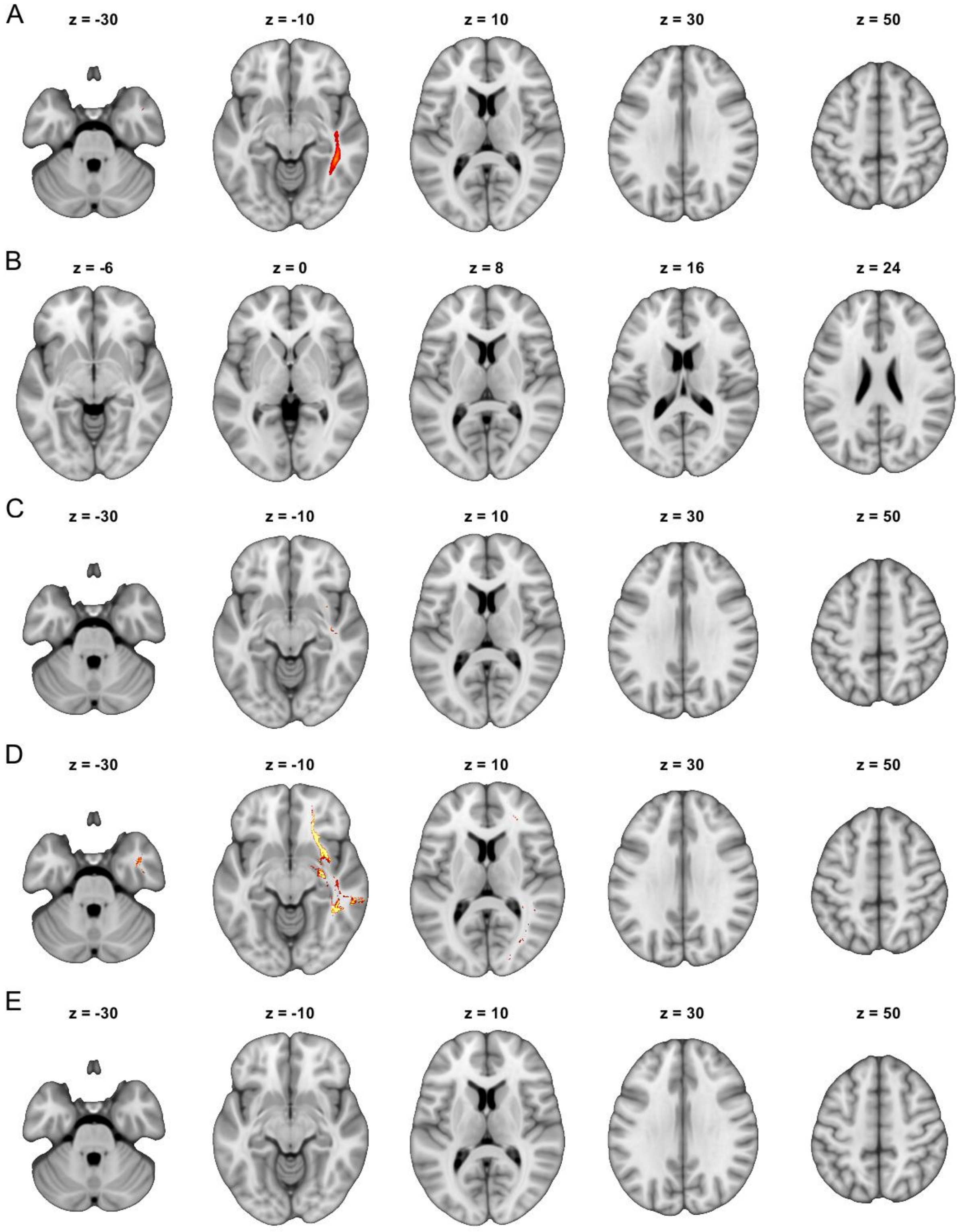

SI Figure 21. Technical validation of structural disconnection mapping for Inferior longitudinal fasciculus R. Please consult legend of Figure 4 in the manuscript for explainations.

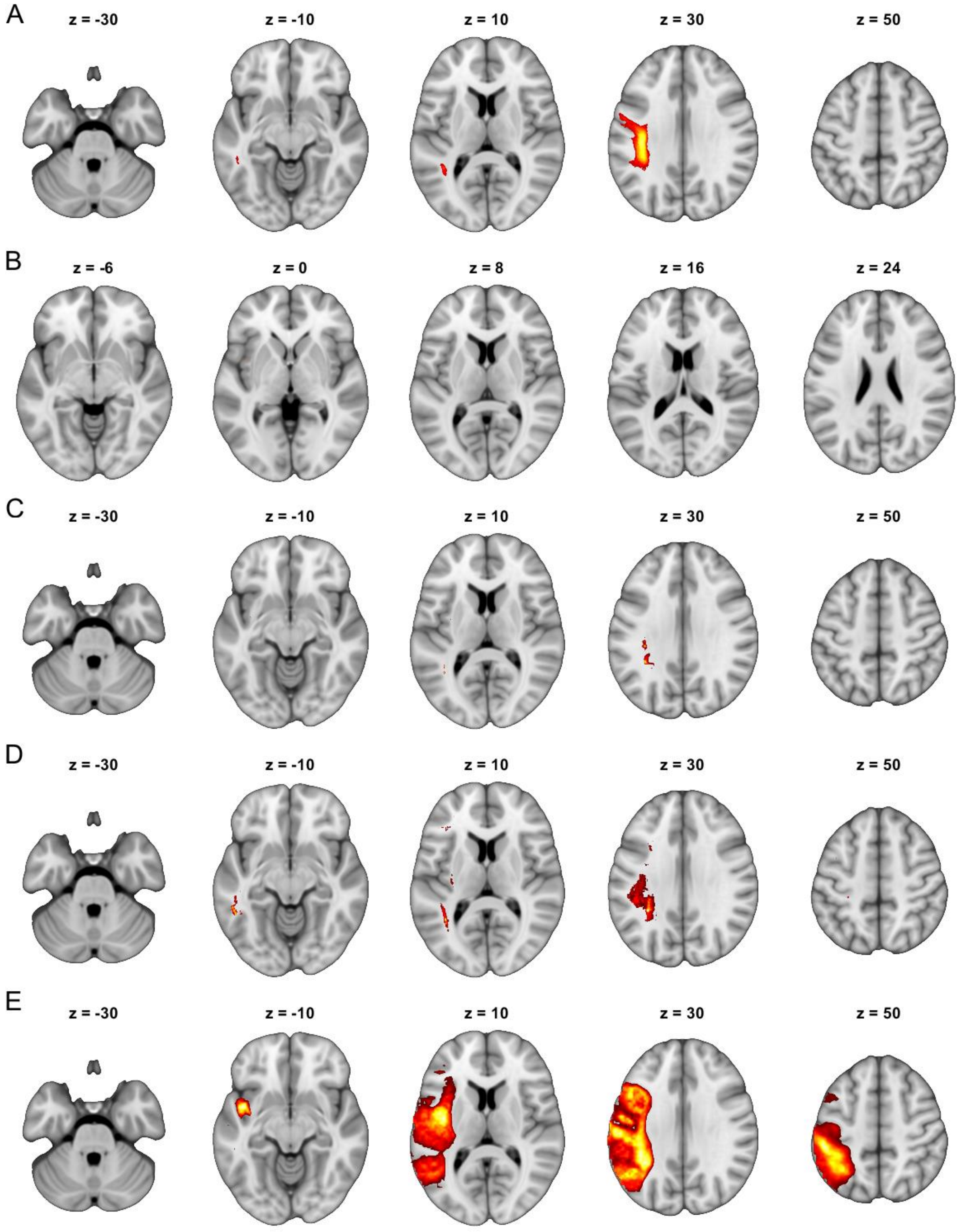

SI Figure 22. Technical validation of structural disconnection mapping for Superior longitudinal fasciculus L. Please consult legend of Figure 4 in the manuscript for explainations.

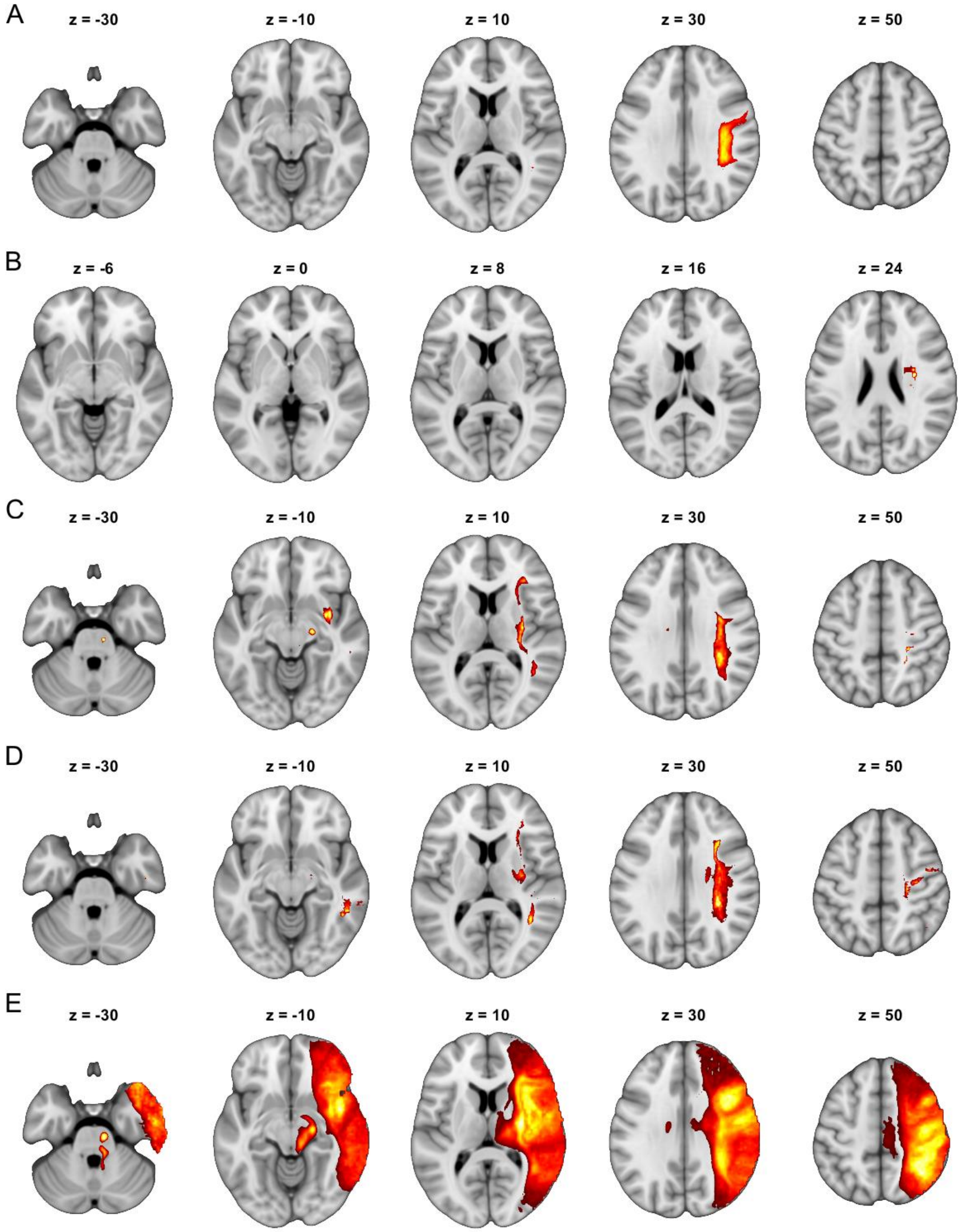

SI Figure 23. Technical validation of structural disconnection mapping for Superior longitudinal fasciculus R. Please consult legend of Figure 4 in the manuscript for explainations.

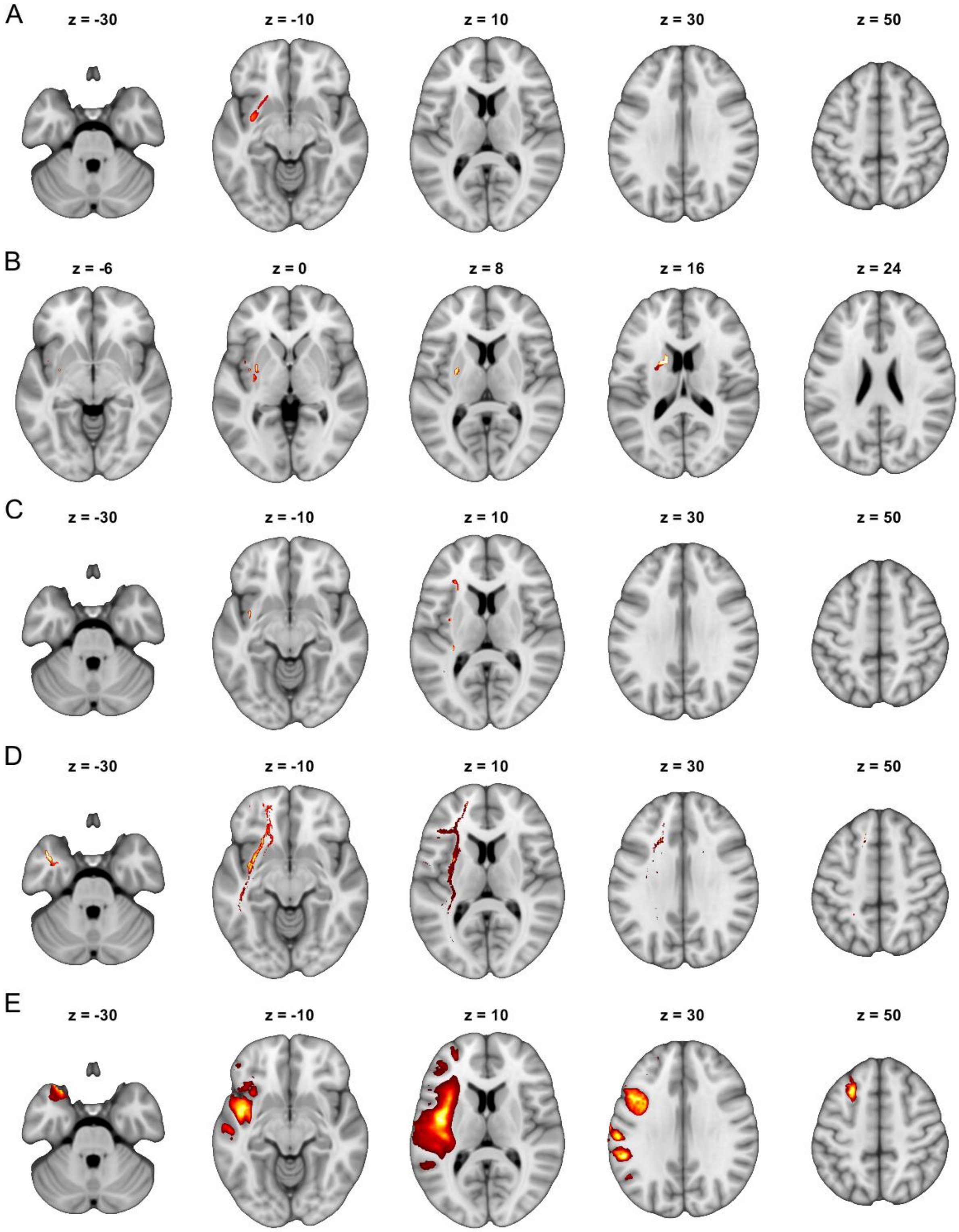

SI Figure 24. Technical validation of structural disconnection mapping for Uncinate fasciculus L. Please consult legend of Figure 4 in the manuscript for explainations.

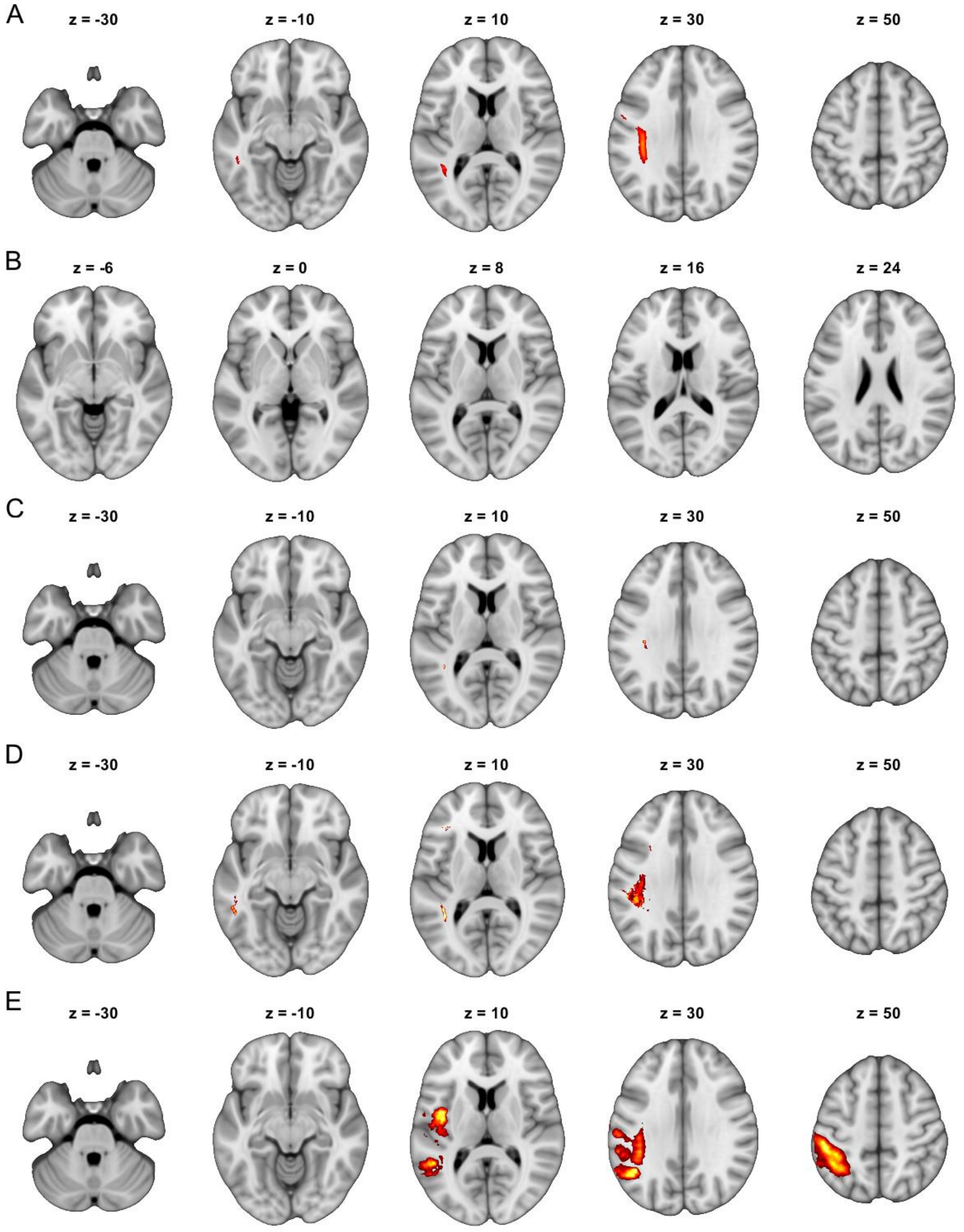

SI Figure 25. Technical validation of structural disconnection mapping for Superior longitudinal fasciculus (temporal part) L. Please consult legend of Figure 4 in the manuscript for explainations.

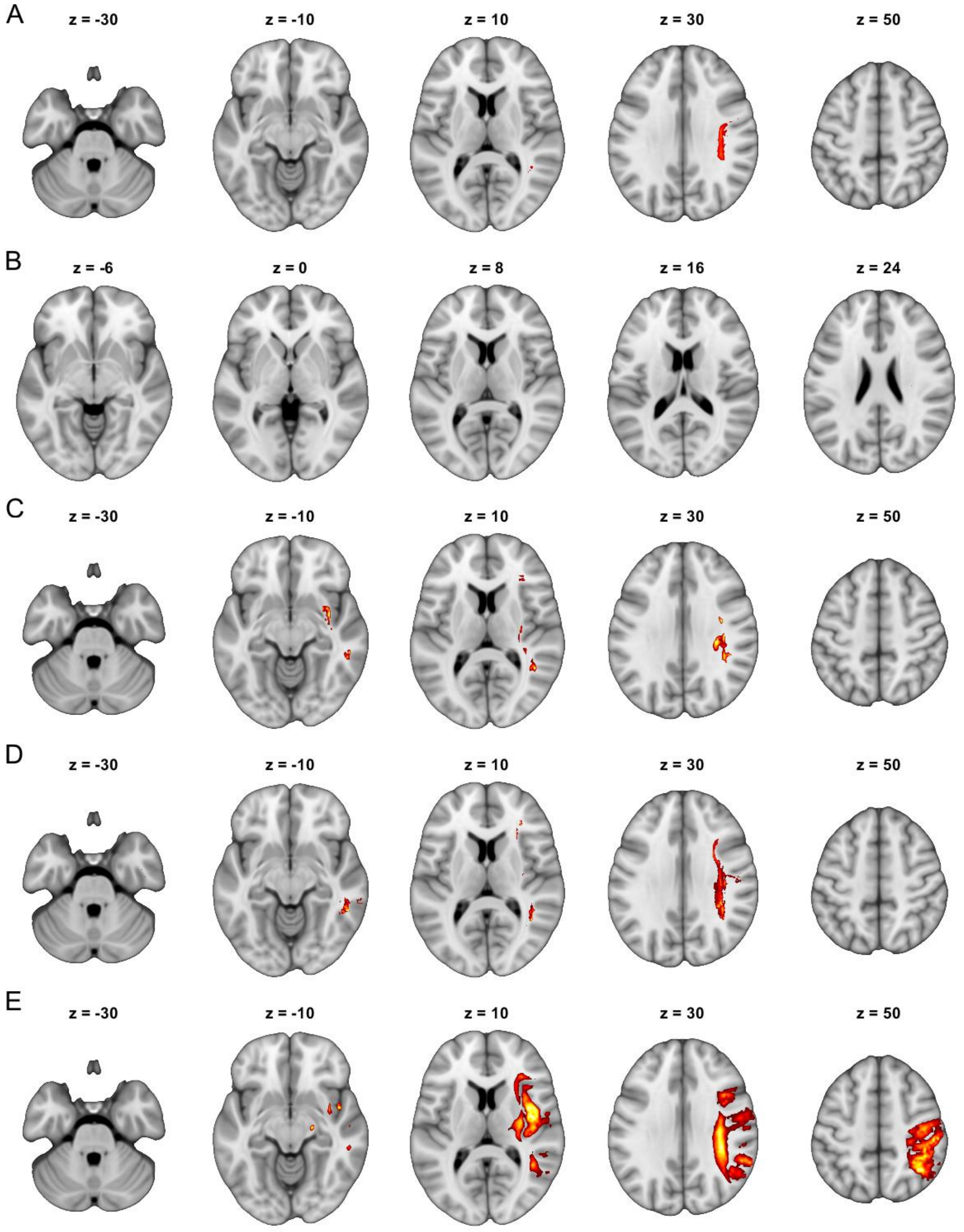

SI Figure 26. Technical validation of structural disconnection mapping for Superior longitudinal fasciculus (temporal part) R. Please consult legend of Figure 4 in the manuscript for explainations.

JHU atlas tract	BCB 10%	BCB 20%	BCB 30%	BCB 40%	BCB 50%	BCB 60%	BCB 70%	BCB 80%	BCB 90%	BCB 100%
Anterior thalamic radiation L	12%	31%	42%	48%	**52%**	51%	47%	39%	28%	14%
Anterior thalamic radiation R	6%	19%	22%	**23%**	21%	18%	12%	7%	2%	0%
Corticospinal tract L	19%	27%	31%	37%	41%	44%	47%	**49%**	48%	42%
Corticospinal tract R	12%	20%	23%	28%	32%	37%	41%	**44%**	**44%**	37%
Forceps major	10%	24%	32%	40%	46%	**49%**	47%	34%	15%	4%
Inferior fronto-occipital fasciculus L	5%	14%	22%	27%	32%	36%	39%	**40%**	**40%**	37%
Inferior fronto-occipital fasciculus R	3%	9%	15%	21%	28%	32%	**35%**	**35%**	**35%**	27%
Inferior longitudinal fasciculus L	4%	8%	12%	14%	16%	**19%**	18%	18%	**19%**	12%
Inferior longitudinal fasciculus R	1%	5%	10%	18%	23%	**25%**	**25%**	23%	19%	8%
Superior longitudinal fasciculus L	12%	24%	33%	38%	**39%**	**39%**	**39%**	36%	29%	12%
Superior longitudinal fasciculus R	12%	18%	23%	28%	32%	**34%**	**34%**	**34%**	31%	26%
Uncinate fasciculus L	1%	3%	4%	4%	5%	6%	8%	10%	**13%**	12%
Superior longitudinal fasciculus (temporal part) L	6%	21%	34%	38%	40%	**41%**	39%	36%	28%	19%
Superior longitudinal fasciculus (temporal part) R	3%	9%	17%	23%	27%	31%	32%	37%	38%	**41%**
mean	*8%*	*17%*	*23%*	*28%*	*31%*	***33%***	***33%***	*32%*	*28%*	21%

SI Table 2. Dice coefficients – technical validation (BCB threshold). This table displays similarity between the atlas tract used to simulate the symptom and the results from analyses based on BCBtoolkit using different thresholds. Higher values imply higher similarity. Abbreviations: BCB x%: disconnection mapping based on tractograms obtained with BCBtoolkit and binarized at x% overlap.

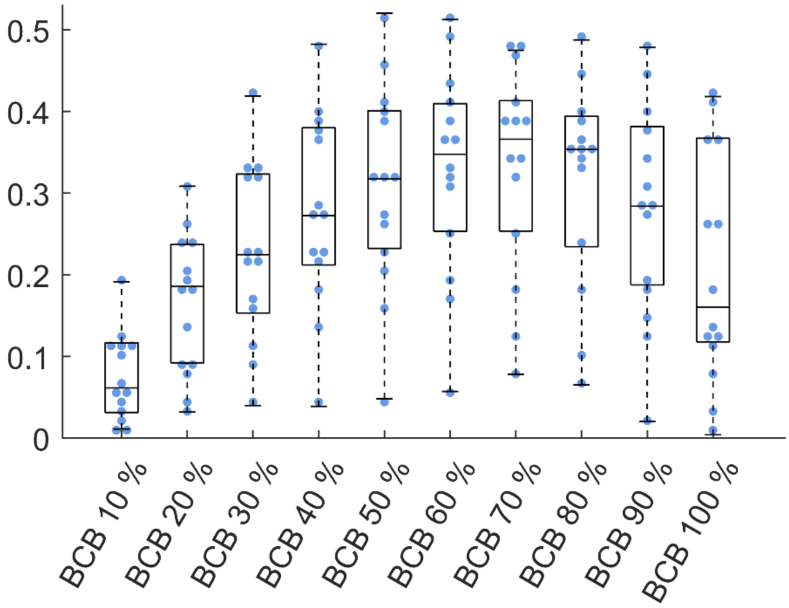

SI Figure 27. Dice coefficients – technical validation (BCB threshold). This boxplot displays similarity (Dice coefficients) between the 14 atlas tracts used to simulate symptoms and the results from analyses based on BCBtoolkit using different thresholds. Higher values imply higher similarity. Central marks of the box represent the median value, the edges are the 25th and 75th percentiles, and the whiskers extend to the most extreme data points not more than 150% of the interquartile range beyond the boxes. Filled circles represent Dice coefficients for individual tracts. Abbreviations: BCB x%: disconnection mapping based on tractograms obtained with BCBToolkit and binarized at x% overlap (blue).

Symptom/JHU atlas tract	BCB 50%	BCB 60%	BCB 70%
Right hemiparesis – left CST	27%	32%	**34%**
Left hemiparesis – right CST	10%	12%	**15%**
mean	*19%*	*22%*	***24%***

SI Table 3. Dice coefficients – clinical validation (BCB threshold). This table displays similarity between the CST and results from disconnection mapping analyses based on BCBtoolkit using different thresholds regarding contralateral hemiparesis in 316 stroke patients. Higher values imply higher similarity. Abbreviations: BCB x%: disconnection mapping based on tractograms obtained with BCBToolkit and binarized at x% overlap.

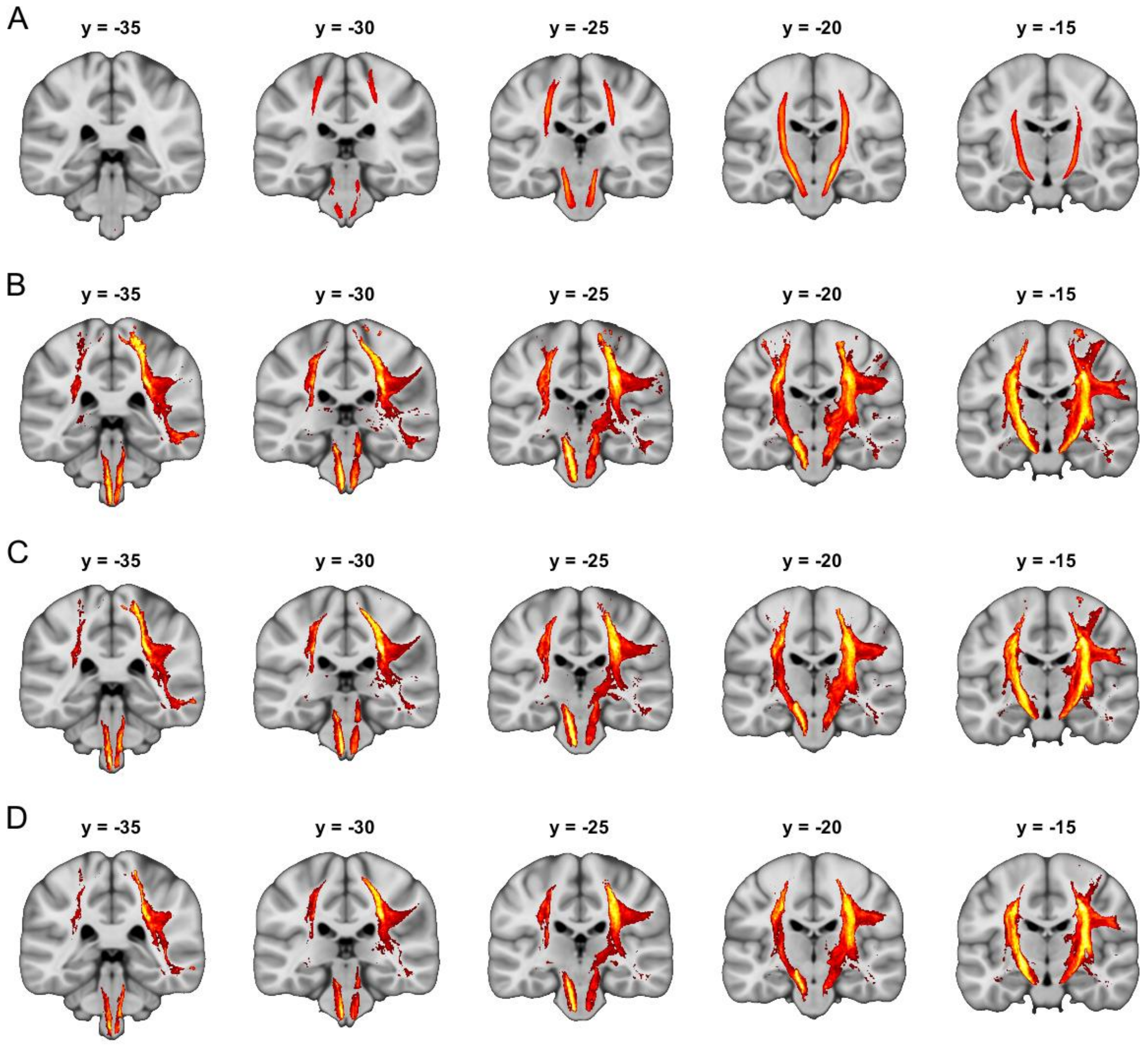

SI Figure 28. Clinical validation of disconnection mapping (BCB threshold). Panel A displays the right and left corticospinal tract taken from the JHU white-matter tractography atlas (thresholded at 25 % overlap). Panel B-D show disconnection mapping analyses based on data of 316 patients with regard to the presence of a left or right sided hemiparesis. Disconnection mapping was based on BCBtoolkit using different thresholds of (A) 50 %, (B) 60 % and (C) 70 %. Left and right sided hemiparesis were analyzed separately and the results were combined in one figure for display purposes. All analyses in (B)-(D) are based on random permutation tests using Freedman-Lane procedure with 4,000 random permutations of the symptom label and lesion volume serving as a covariate of no interest restricted to regions with an overlap of at least 5 lesions/disconnection maps. All maps are thresholded at p(FWE) < 0.05 on the voxel-level. Coordinates refer to MNI space. Left hemisphere is displayed left.

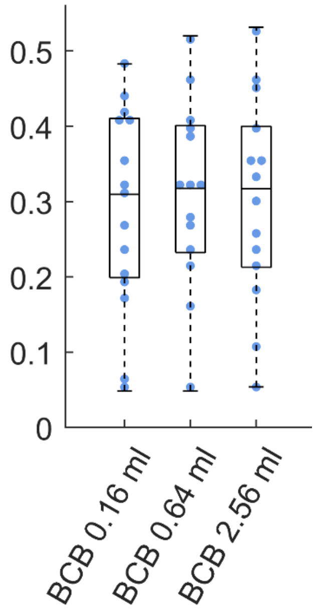

SI Figure 29. Influence of volumetric threshold for simulating symptoms. We repeated the technical validation analysis using the disconnectome maps calculated with BCBtoolkit (binarized at ≥ 50 %). Symptoms were simulated based on thresholds of 0.16 ml, 0.64 ml and 2.56 ml overlap between individual lesion and atlas tracts. Dice scores across all tracts were very similar (paired t-test between all pairs of analyses: $p > 0.50$). The results in the technical validation analyses therefore seem not to critically depend on the exact choice of volumetric threshold for symptom simulation. Central marks of the box represent the median Dice index, the edges are the 25th and 75th percentiles, and the whiskers extend to the most extreme data points not more than 150% of the interquartile range beyond the boxes. Filled circles represent Dice coefficients for individual tracts.
